# Supplementary material for: Sirenian genomes illuminate the evolution of fully aquatic species within the mammalian superorder afrotheria
Source: Nat Commun. 2024 Jul 2;15:5568. doi: 10.1038/s41467-024-49769-x (PMC11219930; doi:10.1038/s41467-024-49769-x)
Supplement: Supplementary file 1 — Supplementary Information [file 41467_2024_49769_MOESM1_ESM.pdf]

Supplementary Information for:

Sirenian genomes illuminate the evolution of fully aquatic species within the mammalian superorder Afrotheria

Ran Tian, Yaolei Zhang, Hui Kang, Fan Zhang, Zhihong Jin, Jiahao Wang, Peijun Zhang, Xuming Zhou, Janet M. Lanyon, Helen L. Sneath, Lucy Woolford, Guangyi Fan, Songhai Li, Inge Seim

SUPPLEMENTARY NOTES 2
Supplementary Note 1. Generation of a chromosome-level dugong reference genome 2
Supplementary Note 2. The enigmatic phylogeny of paenungulates 3
Supplementary Note 3. Molecular evolution of the sirenian cardiovascular system 5
Supplementary Note 4. Sirenian herbivory 6
Supplementary Note 5. Molecular evolution of the sirenian integumentary system 8
Supplementary Note 6. Convergent loss of *PON1* and *CES3* 10
Supplementary Note 7. Inbreeding of dugongs on the Queensland coast 11
SUPPLEMENTARY FIGURES 12
Figure S1 | Overview of extant sirenian taxa 12
Figure S2 | Overview of dugon reference assembly Ddugon_BGI 13
Figure S3 | ML phylogenetic trees of afrotherians inferred using nuclear DNA markers ... 14
Figure S4 | Histology of West Indian manatee flank skin 15
Figure S5 | Stronger binding ability of dugong PER2 with its CRY1 compared to Asian elephant and human forms 16
Figure S6 | Overview of gene inactivation substitutions in sirenian paraoxonase (*PON1*) . 17
Figure S7 | Overview of carboxylesterase 3 (*CES3*) gene inactivation substitutions in sirenians, cetaceans, and pinniped family Phocidae 18
Figure S8 | Neighbor-joining tree of dugongs on the Queensland coast 19
Figure S9 | Population diversity of dugong populations on the Queensland coast 20
Figure S10 | Optimal migration number for TreeMix analysis and gene flow inference from *D*-statistics 21
Figure S11 | Multiple sequence alignment of showing an amino acid substitution in CLPX unique to dugong populations on the northern Queensland coast 22
Figure S12 | Effective population size history of individual dugongs 23
SUPPLEMENTARY TABLES 24
Table S1 | Summary of Ddugon_BGI sequencing and annotation 24
Table S2 | Comparison of assembly quality between afrotherians genome assemblies 25
Table S3 | BUSCO evaluation of afrotherian genome assemblies 26
Table S4 | BUSCO evaluation of afrotherian gene sets 27
Table S5 | STRING literature mining enrichment of genes with inactivating mutations in sirenians 28
Table S6 | List of genes under selective sweep between dugong populations north and south of the Whitsundays Islands. 29

SUPPLEMENTARY NOTES

Supplementary Note 1. Generation of a chromosome-level dugong reference genome

We produced a 3.06 Gb chromosome-level reference assembly (Ddugon_BGI) with the expected [1] 25 pairs of chromosomes (2*n*=50) (**Figure S2**) from a female dugong by combining stLFR and Hi-C sequencing data (**Table S1**), leaving 13.35 Mb (0.44%) unplaced scaffolds. We annotated 18,663 protein-coding genes (**Table S1**) in our assembly.

There are currently (March 2024) four publicly available dugong assemblies. These are a short-read sequencing assembly generated by the Japanese National Institute for Environmental Studies (NCBI Assembly GCA_015147995.1), a synthetic long-read assembly generated using 10x Genomics Chromium technology (hereafter: Ddugon_MaxPlanck) [2], a chromosome-level assembly generated by the DNA Zoo consortium after scaffolding an in-house short-read sequencing assembly (hereafter, Ddugon_DNAzoo), and chromosome-level from the Vertebrate Genome Project (VGP) generated by long-read PacBio HiFi and Omni-C sequencing [3,4] (NCBI Assembly GCA_030035595.1).

Two West Indian manatee (*Trichechus manatus*) assemblies from the same individual are available (**Table S2**). An assembly generated by Foote and colleagues in 2015 [5] recently scaffolded into a chromosome-level assembly by the DNA Zoo consortium (scaffold and contig N50 143.72 Mb and 37.75 kb) was employed in this manuscript. A draft genome [6] (H_Gigas_1.0 in **Table S2**) and a pseudo-genome [2] (generated by mapping short reads to assembly Ddugon_MaxPlanck) of the extinct cold-adapted Steller's sea cow (*Hydrodamalis gigas*) are available but are derived from centuries-old DNA samples and thus highly fragmented, and were not employed in the phylogenomic and genome-scale comparative analyses in this manuscript.

Supplementary Note 2. The enigmatic phylogeny of paenungulates

It is accepted that the closest living relatives to sirenians are the other afrotherian herbivores, the terrestrial proboscideans (elephantids) and hyracoids (hyraxes). They are grouped within the superorder Paenungulata ('almost ungulates' in Latin; now a misnomer as afrotherians are distantly related to ungulates). Their internal topology remains debated. Some studies group proboscideans and sirenians into Tethytheria to suggest a shared origin near the Tethys Sea between Africa and Eurasia before the continents connected, others group hyracoids and proboscideans, or hyracoids and sirenians [7-10]. Morphology (large size and near-hairlessness) may suggest that proboscideans and sirenians should be exclusively grouped. However, these features may mask the true relationships: although extant hyracoids are small (body weight <5kg), their ancestors had a body mass similar to their contemporary proboscideans and sirenians [11]. Furthermore, while it is not known if the shared ancestor of furred modern hyraxes and near-hairless elephants and sirenians had abundant body hair or not, wooly mammoths illustrate that gain of fur from a near-hairless ancestor can be achieved by paenungulates [12,13].

Our phylogenetic analyses based on nuclear DNA (coding regions, 4-fold degenerate sites, and the third codon positions 7,695 protein-coding genes; 5,508 single-copy BUSCO genes; and 627,279 conserved non-exonic elements) supported a sister group relationship between Sirenia with Hyracoidea + Proboscidea (**Figure S1c** and **Figure S3**). This phylogeny agrees with one generated from ultraconserved sites evolving neutrally (or near-neutrally) in coding and non-coding genome regions of 242 placental mammals, including the West Indian manatee, rock hyrax, and African savanna elephant (*Loxodonta africana*) [14] and a recent study employing 98 exonic markers [15]. However, despite strong support, our tree is not necessarily correct. Because paenungulates radiated within a relatively short time, it has been reasoned [e.g., see [16]] that ancient paenungulates were subjected to incomplete lineage sorting (ILS) and introgressive hybridization (also known as introgression). These processes can blur species boundaries and introduce phylogenetic noise. To address this problem, intragenic indels and retroelements (LINEs, SINEs, and LTRs) have been championed as near homoplasy-free phylogenetic markers since there is a low probability that they arose in two species independently and their gain/loss is usually permanent (retroelements, in particular, given their mode of insertion) [17-20]. A recent study [21] considered whole-genome sequences and ≥50-bp intragenic indels and provided support for proboscideans + sirenians (406 indels) but also proboscideans + hyracoids (238 indels; i.e., consistent with our topology in **Figure S1c**) to indicate ILS and possibly also introgression between paenungulates, showing the

ongoing difficulty in resolving their phylogeny. Using our chromosome-level dugong genome as the reference species, we could not reliably call retroelement markers in afrotherian genomes using a recently developed approach that requires 2 kb of sequence flanking both sides of a retrotransposon [19]. The genomes of afrotherian species are currently at various stages of completion and many lack chromosome-level assemblies generated in concert with long-read or synthetic long-read data. Therefore, most assemblies examined here were highly fragmented and contain numerous short contigs (**Table S2**) that may not resolve retroelements. However, near-complete genomes of all afrotherians will likely become available within the next few years, mainly via the Vertebrate Genome Project (VGP) [3]. Regardless of the true relationship between paenungulates, we provide a phylogenetic framework suitable for understanding the evolution of the only fully aquatic species within Afrotheria.

Supplementary Note 3. Molecular evolution of the sirenian cardiovascular system

Sirenians forage in relatively shallow waters and perform short (typically a few minutes) but frequent dives [22]. Although, perhaps not as striking as that of cetaceans, the sirenian cardiovascular system can also sustain conditions that would prove dangerous or deadly to their terrestrial relatives. For example, the Amazonian manatee does not dramatically reduce its heart rate during foraging but shows a relatively moderate 40-50 beats per minute both during dives and at the sea surface [23].

We identified rapid evolution of *SERPINE2* (also known as PN-1 or protease nexin-1), a gene with an emerging role in the cardiovascular system, from cardiac fibrosis to protection against arteriosclerosis and aneurysms [24] (**Supplementary Data 3**). *SCN5A* (cardiac Nav1.5 channel α subunit) and *KCNE2* (potassium voltage-gated channel subfamily E regulatory subunit 2) associated with heart rhythm disturbances are rapidly evolving in sirenians. Human mutations in *KCNE2* (also known as LQT6) result in long QT syndrome (LQTS) [25]. The QT interval is an electrocardiographic measurement that measures the time taken by the cardiac ventricles to depolarize and repolarize at every heartbeat, and its prolongation or shortening is associated with morbidity and mortality [25]. Two sirenian-specific amino acid changes in KCNE2 were observed (**Figure 2d** and **Supplementary Data 5**). The West Indian manatee has a prolonged QT interval compared to Asian and African elephants (*Elephas maximus* and *Loxodonta* spp., respectively) [26,27]. However, like cetaceans and pinnipeds, the sirenian cardiovascular system has many unique anatomical features compared to their closest terrestrial relatives and a direct comparison remains challenging [26,27]. Thus, although sirenian-specific *KCNE2* amino acid substitutions may be damaging in a human or elephant, such effects on the cardiovascular system may be less severe (or even adaptive) in a species with a cardiac anatomy modified for aquatic life. Instead, we propose that the *KCNE2* substitutions are associated with alterations to sirenians' unique diet by serving as a critical constituent of iodide transport (see Nutrient uptake of fully aquatic herbivores section in the main text).

Supplementary Note 4. Sirenian herbivory

Although sirenians can occasionally consume invertebrates, they rely primarily on aquatic plants, including marine seagrasses whose terrestrial ancestors independently returned to the sea during the Cretaceous period, from ~66 to ~100 Mya [22,28]. Whilst dugongs are obligate, specialist seagrass grazers, all three species of manatee are more catholic feeders, taking a range of aquatic and semi-aquatic plant species, and in some cases, may consume emergent grassland species [22]. Adult sirenians consume 10-15% of their body weight daily [22,29]. Similar to the distantly related elephants and hyraxes and some more distantly related mammals (e.g., rhinoceroses), sirenians are hindgut fermenters [22]. Manatees and the dugong have specialized mouthparts [30,31], long and capacious digestive tracts, and prolonged food retention times (up to a week in the dugong) [32]. These modifications, coupled with unique symbiotic microbes [33], presumably maximize yield from nutrient-poor seagrass.

The lipid transporter *ABCG8*, a gene essential for transport of dietary cholesterol, is inactivated in the dugong and West Indian manatee. A recent study comparing 60 mammals, including non-herbivorous afrotherians, reported *ABCG8* loss in the West Indian manatee and elephants [34]. We also confirm a loss of chitinase 5 (*CHIA5*) [35], a gene essential for the digestion of insect and crustacean exoskeletons, in the West Indian manatee and dugong. *ANPEP* (aminopeptidase N) is under positive selection in sirenians (**Supplementary Data 1**). It encodes an enzyme that finalizes the digestion of peptides after their hydrolysis in the intestines, brain, and other cells and tissues. *ANPEP* was previously reported to be under positive selection in sirenians (West Indian manatee), cetaceans (bottlenose dolphin, *Tursiops truncatus*, and killer whale, *Orcinus orca*), and pinnipeds (walrus, *Odobenus rosmarus*) and hypothesized to play a role in reducing oxidative stress in marine mammals by adjusting cysteine and glycine availability in the glutathione metabolism pathway [5]. This gene is also under positive selection in the ancestral branch of the dietary-diverse bat family Phyllostomidae [36] and has lower enzyme activity in herbivorous bird [37] and rodent [38] species. We speculate that these observations hint at modifications of this digestive enzyme in marine mammals consistent with their dietary substrates, in addition to a role in oxidative stress resistance.

Nearshore marine plants and aquatic plants from rivers and swamps have high levels of iodine. A study of captive West Indian manatees found that switching their diet from terrestrial plants to seagrass over 19 days increased blood thyroid hormone levels [39] – as expected given the difference in dietary iodine content. Wild manatees showed higher levels

of circulating thyroid hormones than any captive diet group [39], which we hypothesize resulted from a lifetime on a high-iodine seagrass diet and associated energy metabolism balance.

Supplementary Note 5. Molecular evolution of the sirenian integumentary system

The integumentary system, the skin and its appendages, is the largest organ of the mammalian body and a barrier to the external environment that maintains water balance and contributes to thermoregulation [40]. Recent studies highlight skin adaptations since the sirenian and elephantid ancestors diverged (~58 Mya; **Figure S1c**). Elephantid species acquired novel gene changes associated with skin modifications to life on the arid African continent [41] and in the Arctic [12]. Sirenians adapted their skin to life in water, with the extinct Steller's sea cow further evolving a larger body size, thicker blubber, and thick, rough skin [2] to adjust to the sub-Arctic environment. The integumentary system of manatees and the dugong is quite similar. Exceptions include their epidermis and blubber [42,43]. The manatee epidermis is structurally more similar to cetaceans (black in color), while the dugong epidermis is brown-gray. The dugong has a single-blubber layer (except for the centrum), while the manatee has two layers throughout the body [42,43] (**Figure S4**).

Previously reported sirenian epidermal gene loss events include type I keratins [44,45], arachidonic acid 15-lipoxygenase-1 (*ALOX15*) [46], and kallikrein 8 (*KLK8*; also expressed by sweat glands) [47]. We observed *TCHH1* and *TCHHL1* loss, genes highly expressed by the hair follicle and the hair shaft [48] that produce their structural component, trichohyalin. Our analysis and recent studies also identified sirenian loss of hair follicle-associated keratin genes, goose-type lysozyme 2 (*LYG2*), and acyl-CoA wax alcohol acyltransferase 2 (*AWAT2*) [44,45,49,50]. Shared morphological features of sirenians and cetaceans, the only fully aquatic mammals, include an absence of pelage (i.e., coat) hair follicles [51]. Sirenians are not completely hairless, however, but harbor sparsely distributed body hairs that likely serve a tactile function [51,52]. Not surprisingly, many of the same epidermal and dermal genes have been independently lost in cetaceans and sirenians: type I and II keratins (KRTs) [44,45], *ALOX5* [46], *LYG2* [50], *AWAT2* [49], *MCR5* [53,54], and *KLK8* [47]. Four genes identified in our analysis (*TCHH1L*, *AWAT15*, *KRT2*, and *KRT77*) are among eight independently lost in cetaceans and hippopotamuses (hippos), a semi-aquatic sister group to cetaceans [55]. Two recent studies demonstrated a loss of melanocortin receptor 5 (*MC5R*), a gene with sebaceous gland functions, in the West Indian manatee, cetaceans, and other species with no apparent sebaceous glands [53-55].

Most fat in marine mammals is found in their hypodermis (blubber), a skin layer rich in white adipose tissue (WAT) with an inner stratum of brown adipose tissue (BAT) [51,56,57]. The mammalian hypodermis also contains adipose-derived stem cells (ADSC) that give rise to adipocytes of the BAT and WAT. Brown adipocytes of BAT and beige adipocytes of WAT

express uncoupling protein 1 (*UCP1*), a gene essential for non-shivering thermogenesis (NST) [58]. During cold exposure, thyroid hormones regulate thermogenesis directly via *UCP1* [59]. The blood levels of thyroid hormones in the West Indian manatee [39] and a tropical cetacean are similar, while the levels of cold-water cetaceans are approximately twice as high [60]. *UCP1* is reportedly inactivated in many terrestrial mammals, cetaceans, sirenians (including the cold-adapted Steller's sea cow), and some pinnipeds [61-64]. Numerous factors may explain why *UCP1* loss is tolerated in certain mammals. Prominent among these is that species with a large body size are inherently better at conserving heat [63,65]. *UCP1* may only be lost in northern and southern elephant seals (genus *Mirounga*), species with very large body sizes [66-68]. All pinnipeds also have fur, a much better insulator than blubber [65] that may somewhat reduce their reliance on UCP1-mediated thermogenesis. Gene loss may not always abolish function, however [69]. A recent study reported that, despite harboring several ostensibly inactivating mutations, *UCP1* is expressed by cetacean BAT and shows a reduced function that may serve to maintain a balance between maintaining a thick blubber and thermogenesis [56].

Within Afrotheria, inactivating *UCP1* mutations is not unique to sirenians. It is also found in hyraxes (their ancestor had a much larger body size [11]) and elephants, while the smaller species of their insectivorous sister clade (Afroinsectiphilia) have retained the gene and show evidence of *UCP1* neo-functionalization [62,63]. Thus, large-bodied paenungulates, which emerged on the warm African continent, likely later evolved cold-hardiness adaptations that do not rely on *UCP1*-mediated thermogenesis, as illustrated by the larger body size and thick, hyperkeratotic epidermis of the Steller's sea cow [2] and fur of woolly mammoths. While no blubber expression and functional data on *UCP1* is currently available for an extant sirenian, their thin blubber compared to cetaceans and pinnipeds [51], low metabolic rate (at least in manatees), and restriction to tropical and subtropical habitats suggests that the dugong and manatees are naturally susceptible to cold temperatures.

Supplementary Note 6. Convergent loss of *PON1* and *CES3*

While the herbivorous sirenians are at a much lower position in the food web and show relatively low levels of persistent organic pollutants (i.e., organic chemicals that persist in the environment) compared to cetaceans at similar geographic locations [70], they are likely sensitive to certain manmade chemicals.

Loss of *PON1* (paraoxonase 1) in most aquatic mammals – sirenians (including the extinct Steller's sea cow *Hydrodamalis gigas*), cetaceans, pinnipeds, beavers, and the sea otter *Enhydra lutris* – is thought to reduce oxidative stress and associated inflammation induced by diving [71,72]. *PON1* was also identified in our pseudogene analysis, confirming a single shared premature stop codon event in the dugong and West Indian manatee as well as several independent inactivation mutations (**Supplementaray Data 8** and **Figure S6**). The [presumably adaptive] loss of *PON1* likely became maladaptive in aquatic mammals after the industrialization of agriculture as the enzyme protects against organophosphate pesticides (the ozone forms of chlorpyrifos and diazinon) [71].

We identified loss of carboxylesterase 3 (*CES3*; also known as *ES31*) in sirenians (**Supplementary Data 8**), cetaceans (loss of first nine coding exons and downstream inactivating mutations), and phocids (inactivating mutations, including a 10-bp deletion in Phocidae, the largest pinniped family [73]) (**Figure S7**). Loss of *CES3* – by the West Indian manatee and killer whale [46], and by four cetaceans and two hippos [55] – has previously been reported but not discussed. A premature stop codon is shared by all sirenians, while the dugong and Steller's sea cow share an additional stop codon (**Figure S7**). *CES3* and its homolog *CES1* have been associated with macrophage cholesterol ester metabolism and are both also expressed by the liver and intestines, where they break down ester bonds of endogenous compounds and xenobiotics [74-76]. While *CES3* expression is much lower than *CES1* and its enzyme has several magnitudes lower catalytic efficiency than CES1 for many compounds [74-76] (and, thus, *CES3* loss in marine mammals is likely compensated), carboxylesterase 3 may show exclusive specificity against manufactured compounds such as pesticides.

Supplementary Note 7. Inbreeding of dugongs on the Queensland coast

Members from the seven Queensland dugong locations carried a small number of >1Mb runs of homozygosity (median five ROHs) (**Figure S9d,e** and **Supplementary Data 11**). An exception was individuals from Airlie Beach, Whitsunday Islands, the location of the apparent genetic break border. Only three individuals were obtained from this location. Sampling additional individuals is necessary before speculating on inbreeding in this population. Nevertheless, the inbreeding coefficient (F_{ROH} >1Mb) was very low across all Queensland dugongs (median $F_{ROH>1Mb} = 0.0023$) and did not differ significantly between populations (**Figure S9e**) (Kruskal-Wallis $P = 0.25$).

SUPPLEMENTARY FIGURES

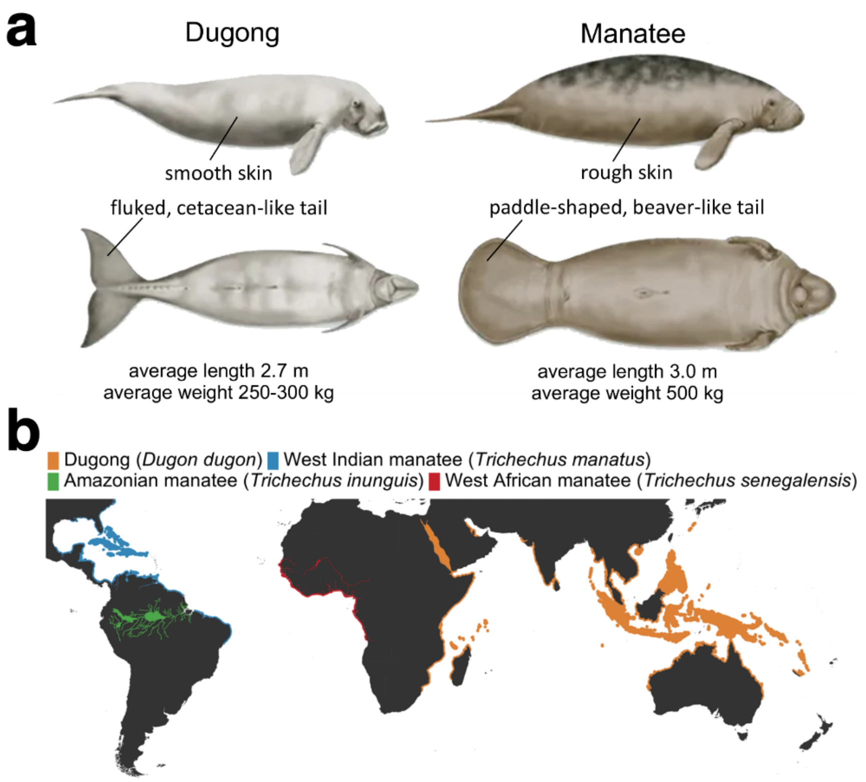

Figure S1 | Overview of extant sirenian taxa

a, Features of extant sirenians. In addition to common sirenian features, the dugong (*Dugong dugon*) and manatees (genus *Trichechus*; here the West Indian manatee, *T. manatus*) show various morphological differences. By courtesy of Encyclopædia Britannica, Inc. **b,** Species distribution of sirenians. Species ranges ranges derived from spatial data on marine mammals from the International Union for the Conservation of Nature Red List of Threatened Species, December 2022 [version 6.3]. https://www.iucnredlist.org; Downloaded on 12 January 2023. Note that the dugong was very recently (2022) declared extinct in Chinese and Japanese waters.

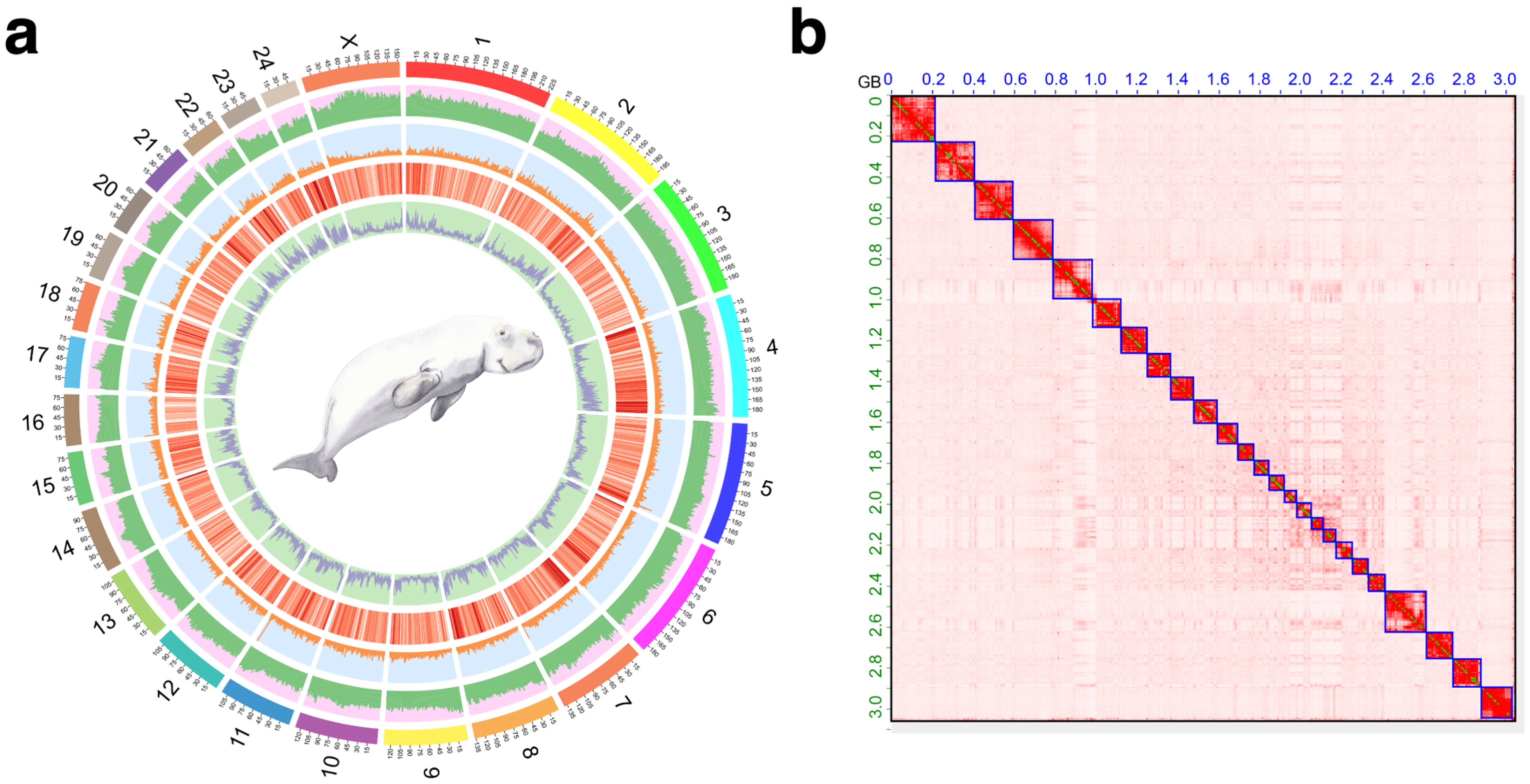

Figure S2 | Overview of dugon reference assembly Ddugon_BGI

a, Circos plot of *Dugong dugon* assembly Ddugon_BGI. The outermost segment represents chromosome sequences, with the numbers on the external surface indicating genome size in Mb. The line plots, from outside to inside, respectively represent the distribution of retrotransposon ratio (from 0.02 to 0.82), DNA transposon ratio (from 0 to 0.08), gene density (from 0 to 54) at 1Mb windows and GC content (from 0.33 to 0.64) at 100 kb windows. Image of dugong courtesy of Liudmila Kopecka/Shutterstock.com. **b,** Genome-wide Hi-C contact matrix of Ddugon_BGI. The heat map shows long-range contacts and scaffolding of the genome assembly. Blue squares denote chromosomes.

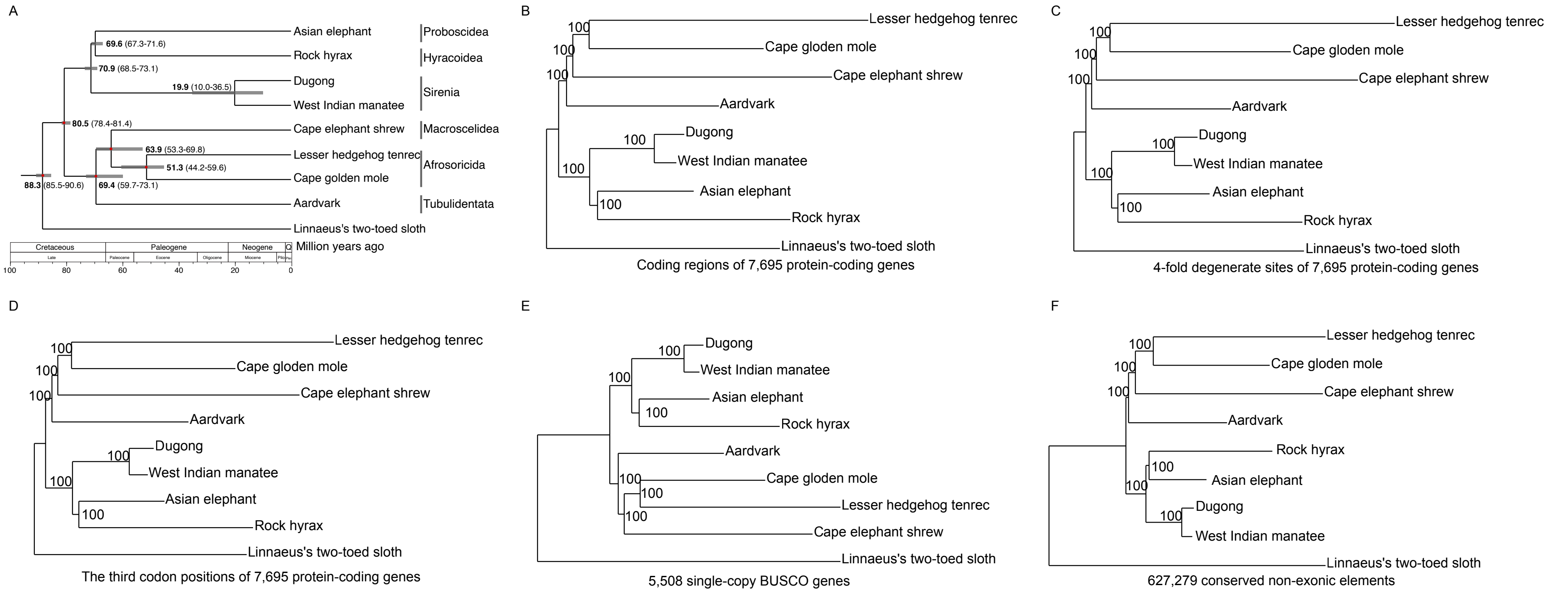

Figure S3 | ML phylogenetic trees of afrotherians inferred using nuclear DNA markers

a, Maximum likelihood (ML) phylogenetic tree from the coding sequences of 7,695 orthologs from eight afrotherian species, rooted with a xenarthran outgroup (the Linnaeus's two-toed sloth). 1,000 bootstraps were used, and all nodes have 100% support. The number in each node represents the divergence time among species a red circle indicates the fossil record used for calibration in the node. The gray bars on the nodes indicate the 95% credibility intervals of the estimated posterior distributions of the divergence times. Afrotherian orders are shown to the right of each representative species. **b,** coding regions of 7,695 protein-coding genes **c,** 4-fold degenerate sites of 7,695 protein-coding genes. **d,** the third codon positions of 7,695 protein-coding genes. **e,** 5,508 single-copy BUSCO genes. **f,** 627,279 conserved non-exonic elements (CNEEs). Trees were concatenated into a single super sequence and a ML phylogenetic tree construed using RAxML. 1,000 bootstrap replicates were performed to compute the node support (100 represents 100% support).

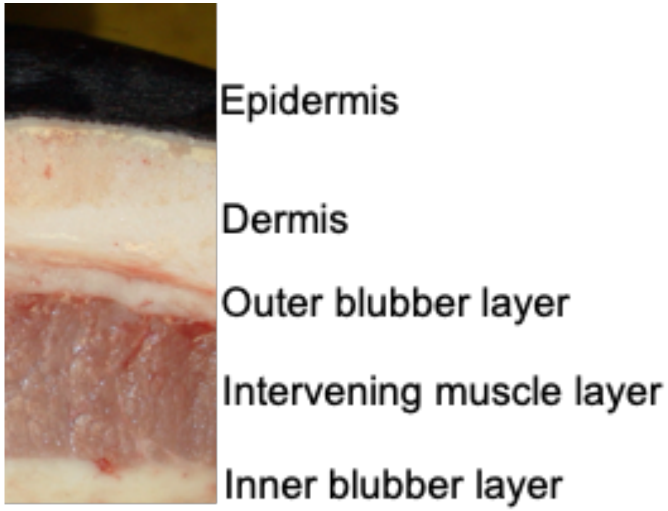

Figure S4 | Histology of West Indian manatee flank skin

Note the thin epidermis (black) and thick dermis. Photo by Michael Lusk (URL: https://www.flickr.com/photos/killkudzu/7619052292) under CC BY-SA 2.0.

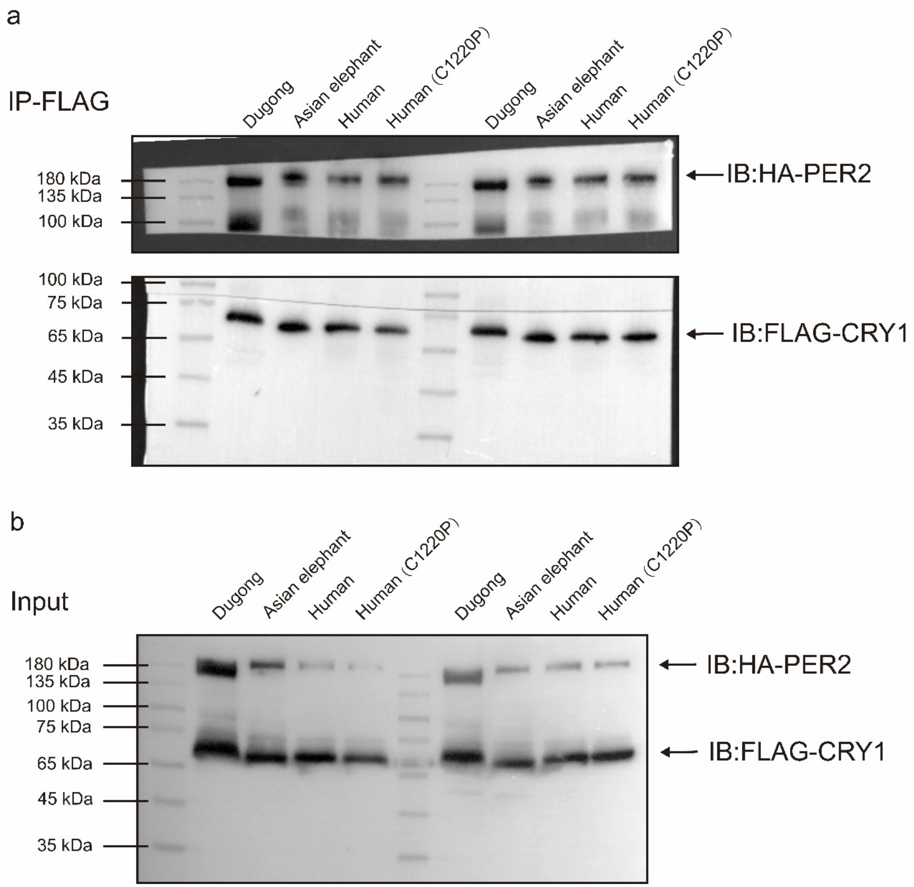

Figure S5 | Stronger binding ability of dugong PER2 with its CRY1 compared to Asian elephant and human forms

Representative western blots depicting **a,** co-immunoprecipitation eluate fractions and **b,** input lysates derived from HEK293T cells expressing *PER2* and *CRY* of dugong, Asian elephant, and human. Human C1220P denotes human *PER2* mutated to match dugong residue 1220 (a proline). The cell lysate was subjected to immunoprecipitation with anti-FLAG magnetic beads followed by immunoblot of HA-PER2 and FLAG-CRY1 (right-hand side arrows). Numbers on the left side of the gels represent the band of a StarRuler 10–180 kDa molecular weight marker shown in each blot's first and sixth lane. Subfigure a, shows the same blot with a different exposure time.

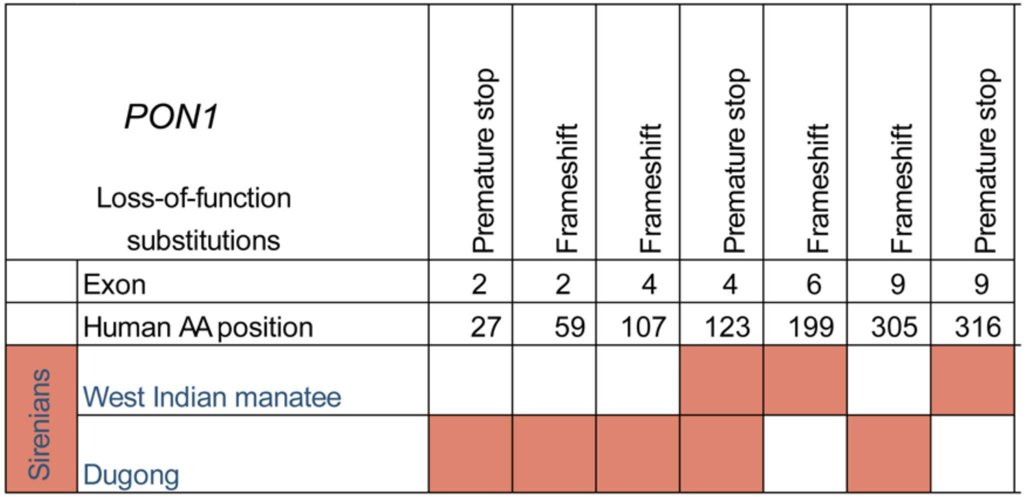

Figure S6 | Overview of gene inactivation substitutions in sirenian paraoxonase (*PON1*)

Colored cells indicate inactivating events. Loss of function substitutions include exon deletions, premature stop codons, and frameshifts.

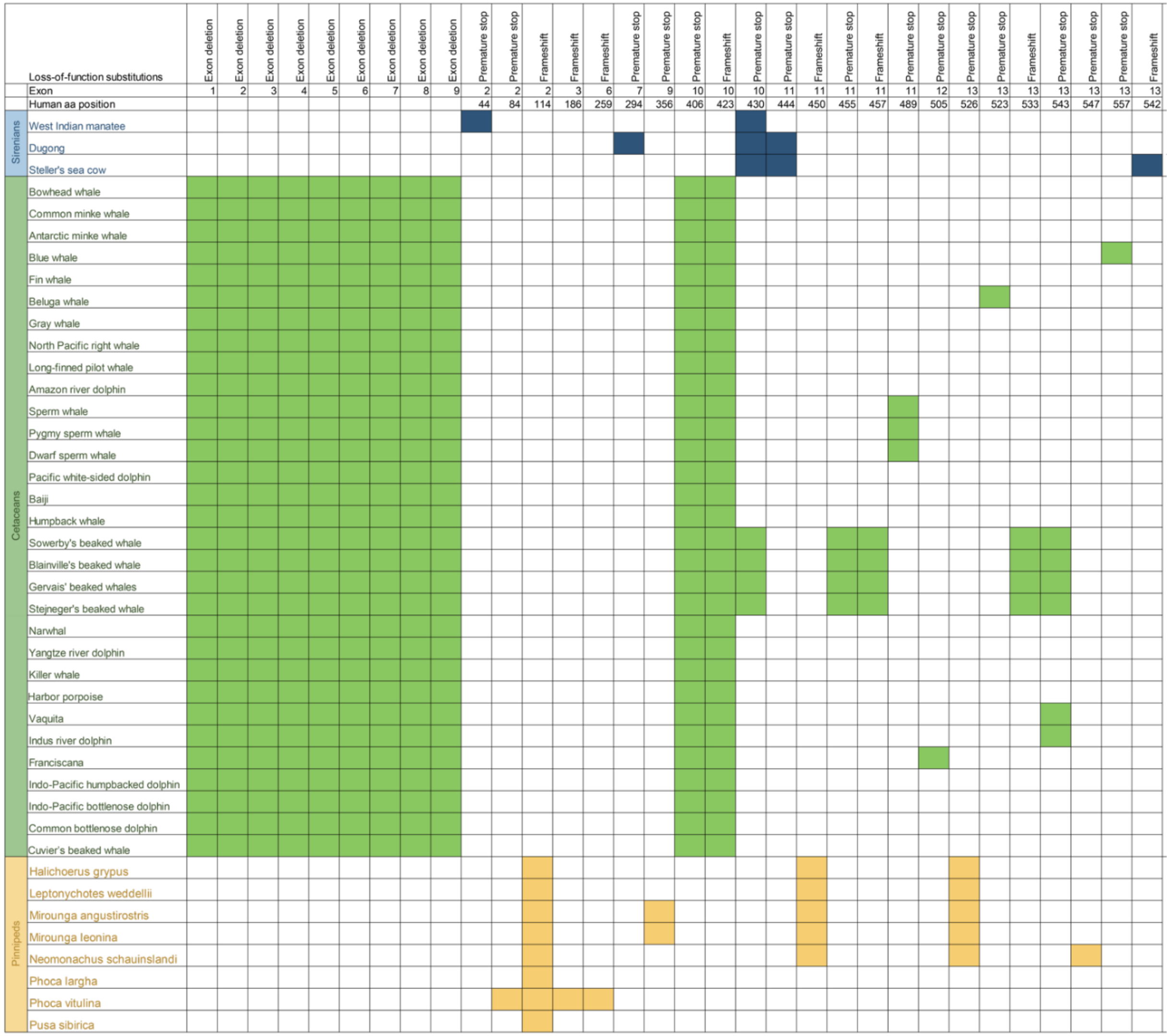

Figure S7 | Overview of carboxylesterase 3 (*CES3*) gene inactivation substitutions in sirenians, cetaceans, and pinniped family Phocidae

Colored cells indicate inactivating events. Loss of function substitutions include exon deletions, premature stop codons, and frameshifts.

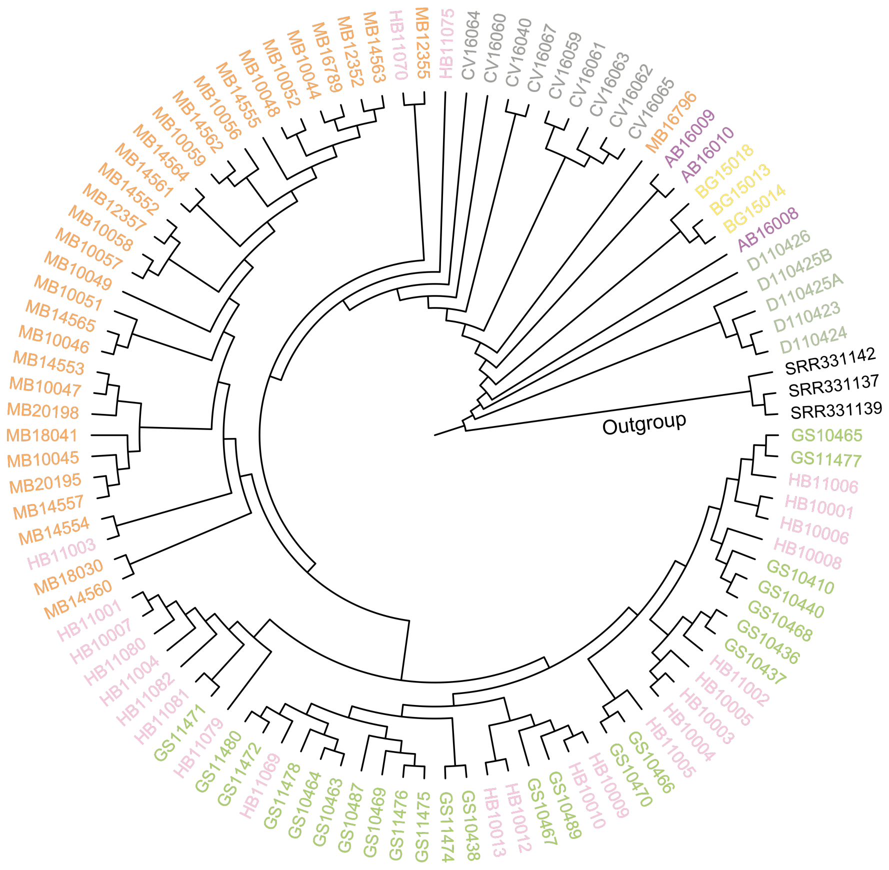

Figure S8 | Neighbor-joining tree of dugongs on the Queensland coast

Neighbor-joining (NJ) tree indicates structure among seven dugong populations on the Australian east coast, supporting a break between population north and south of the Whitsunday Islands. Torres Strait individuals in the NJ tree (c) have a 'D' prefix. The West Indian manatee was used as an outgroup (black font).

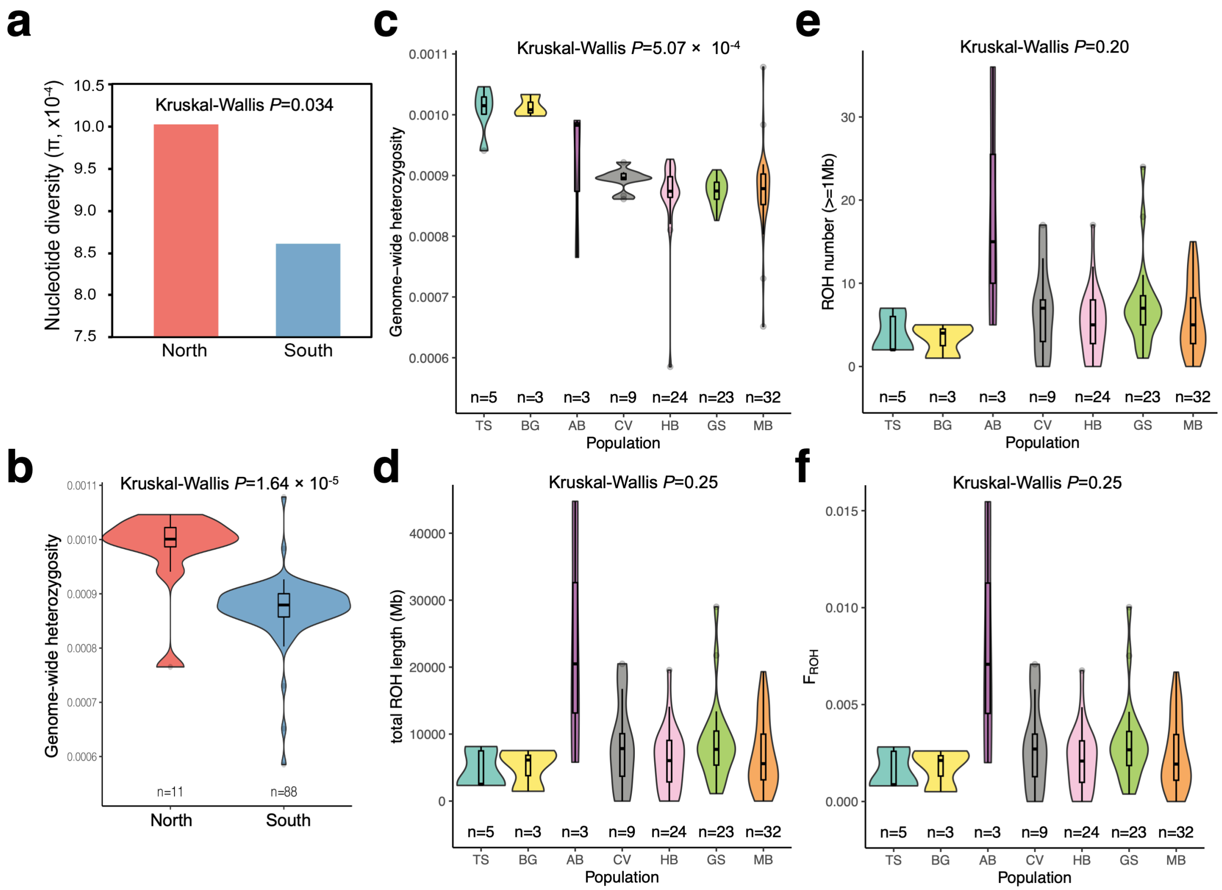

Figure S9 | Population diversity of dugong populations on the Queensland coast

Population diversity of northern and southern groups (a and b) and each subgroup separately (c-f). **a,** relative nucleotide diversity (π) of northern (TS, BG, and AB) and southern (CV, HB, GS, and MB) population groups. Statistics performed on the mean π values of the seven locations. **b,** Genome-wide heterozygosity of northern (TS, BG, and AB) and southern (CV, HB, GS, and MB) population groups. Statistics performed on heterozygosity values of 99 individuals. **c,** Genome-wide heterozygosity. **d,** Number of runs of homozygosity (ROH) larger than 1 Mb. TS denotes Torres Strait, QLD; BG, Bowling Green Bay, QLD; AB, Airlie Beach, QLD; CV, Clairview, QLD; HB, Hervey Bay, QLD; GS, Great Sandy Strait, QLD; MB, Moreton Bay, QLD. **e,** Total lengths of ROHs. **f,** The proportion of the autosomal genome in ROH (F_{ROH} >1Mb).

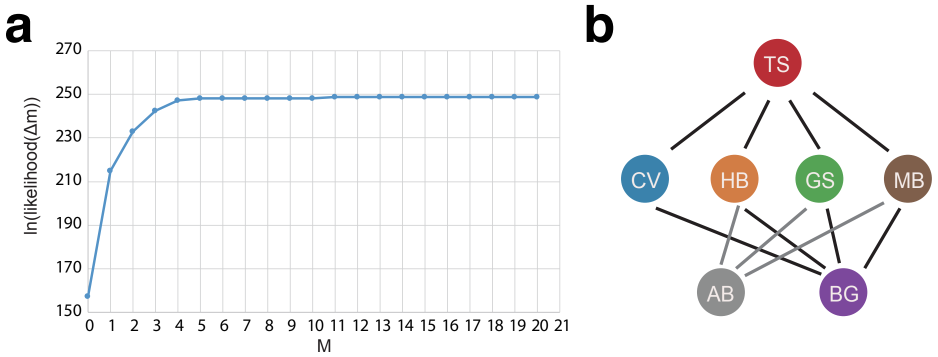

Figure S10 | Optimal migration number for TreeMix analysis and gene flow inference from *D*-statistics

a, Graph of OptM output for 20 migration edges to estimate the optimal number of migration events (*M*) for use in TreeMix analysis.
b, Schematic diagram of gene flow detection using Patterson's *D*-statistic (ABBA BABA statistics; see **Table S15**). Lines indicate gene flow between populations. TS denotes Torres Strait, QLD; BG, Bowling Green Bay, QLD; AB, Airlie Beach, QLD; CV, Clairview, QLD; HB, Hervey Bay, QLD; GS, Great Sandy Strait, QLD; MB, Moreton Bay, QLD.

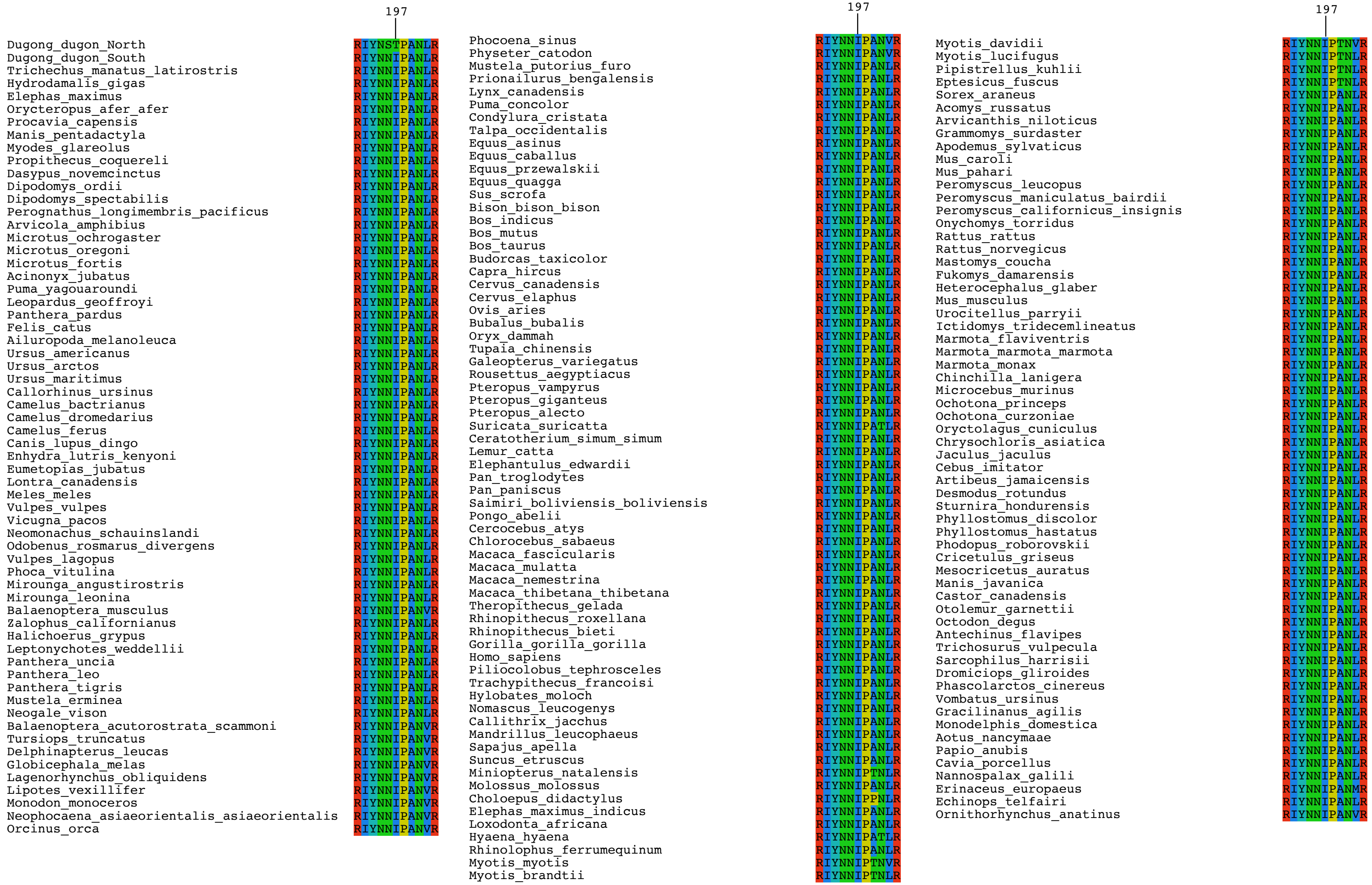

Figure S11 | Multiple sequence alignment of showing an amino acid substitution in CLPX unique to dugong populations on the northern Queensland coast

Trichechus_manatus_latirostris denotes West Indian manatee; Hydrodamalis_gigas, Steller's sea cow.

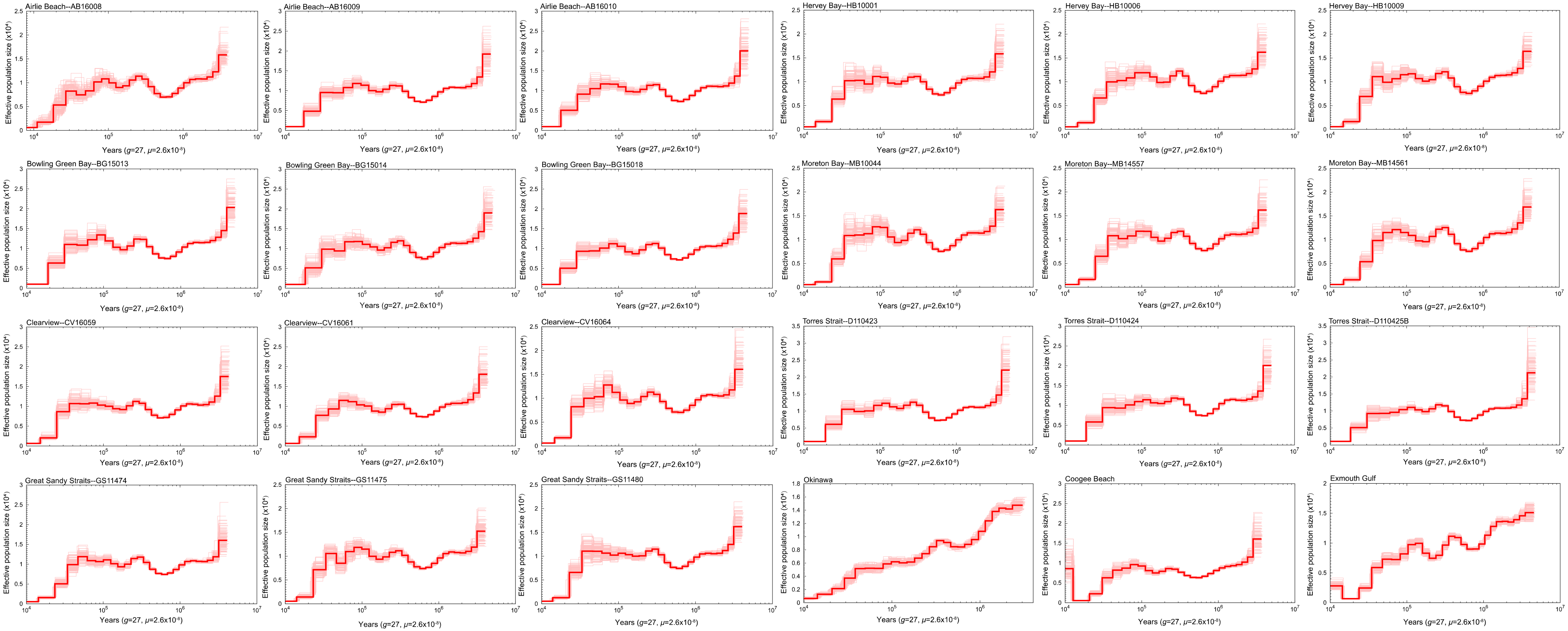

Figure S12 | Effective population size history of individual dugongs

Historical population sizes (N_e) were estimated using the pairwise sequentially Markovian coalescent (PSMC) method and diploid genome sequences (autosomes-only). The *y*-axis shows N_e, with 100 bootstrap estimates indicated by lighter lines. Plots were scaled using a mutation rate (μ) of 2.60×10^{-8} substitutions per nucleotide per generation and species-specific generation times (g) of 27 years. Three random individuals were selected from the Queensland locations Torres Strait, Bowling Green Bay, Airlie Beach, Clairview, Hervey Bay, Great Sandy Strait, and Moreton Bay.

SUPPLEMENTARY TABLES

Table S1 | Summary of Ddugon_BGI sequencing and annotation

Hi-C anchored rate refers the proportion of scaffolded bases assembled onto 25 pseudochromosomes (chr 24 autosomes + chr X).

**Genome assembly**	Assembly size (scaffold)	3.06 Gb
	Assembly size (contig)	2.99 Gb
	Hi-C anchored rate	99.56%
	Contig number	26,850
	Contig N50	226.99 kb
	Longest contig	2.29 Mb
	Scaffold number	2,926
	Scaffold N50	136.91 Mb
	Longest scaffold	228.81 Mb
	GC content	40.58%
	Gaps (N)	2.27%
**Transposable elements**	DNA	1.23%
	LINE	44.92%
	SINE	3.41 %
	LTR	10.44 %
	Unknown	0.0030 %
	Total	49.200%
	Predicted genes	19,897
**Protein-coding genes**	Average transcript length	63,305.31 bp
	Average coding sequence length	1,649.23 bp
	Average exon length	168.60 bp
	Average intron length	7,020.79 bp
	Functionally annotated genes	18,663

Table S2 | Comparison of assembly quality between afrotherians genome assemblies

Ddugon_BGI denotes the assembly generated in our study. Assemblies with the suffix _HiC were generated by DNA Zoo, the remainder are assemblies are available via NCBI. Assemblies GCA_905400935.1, GCA_015147995.1 and Dugong_dugon_HiC are denoted 'Ddugon_MaxPlanck', 'Ddugon_NIES' and 'Ddugon_DNAzoo)' elsewhere in this manuscript. The DNA Zoo assemblies can be found at dnazoo.org/assemblies/Dugong_dugon (Ddugon_HiC), dnazoo.org/assemblies/Trichechus_manatus (TriManLat1.0_HiC), dnazoo.org/assemblies/Elephas_maximus (Elephas_maximus_HiC), dnazoo.org/assemblies/Procavia_capensis (Pcap_2.0_HiC), dnazoo.org/assemblies/Orycteropus_afer (OryAfe1.0_HiC). Please note that the Steller's sea cow (*Hydrodamalis gigas*) was assembled using short-insert reads from ancient DNA samples and is partial assembly of a genome approximately the same size as the dugong and manatee (~3 Gb).

Latin name	Common name	Assembly	Chromosome-level?	Scaffold genome size (Gb)	Contig genome size (Gb)	G+C content (%)	No. scaffolds	No. contigs	Scaffold N50 (Mb)	Contig N50 (kb)
Dugong dugon	Dugong	Ddugon_BGI	Yes	3.06	2.99	40.58	2,926	26,850	136.91	226.99
Dugong dugon	Dugong	Ddugon_HiC	Yes	3.10	3.08	40.63	335,494	391,685	118.74	63.95
Dugong dugon	Dugong	GCA_030035595.1	Yes	3.16	3.16	40.65	198	294	140.67	57,632.67
Dugong dugon	Dugong	GCA_905400935.1	No	3.20	3.10	40.46	16,045	52,168	3.11	155.49
Dugong dugon	Dugong	GCA_015147995.1	No	2.62	2.61	40.73	116,852	285,112	0.031	17.96
Hydrodamalis gigas	Steller's sea cow	H_Gigas_1.0	No	1.24	1.24	44.13	998,083	1,091,214	0.0014	13.45
Trichechus manatus	West Indian manatee	TriManLat1.0_HiC	Yes	3.10	2.77	40.73	5,652	166,508	143.72	37.75
Trichechus manatus	West Indian manatee	ASM3001377v1	No	3.09	3.09	40.66	15,608	15,641	0.49	486.87
Elephas maximus	Asian elephant	Elephas_maximus_HiC	Yes	3.21	3.19	40.83	543,483	602,016	95.96	57.61
Procavia capensis	Rock hyrax	Pcap_2.0_HiC	Yes	3.61	3.20	41.00	59,965	327,292	133.72	35.45
Orycteropus afer	Aardvark	OryAfe1.0_HiC	Yes	4.42	3.42	40.07	22,223	434,556	644.00	17.65
Echinops telfairi	Lesser hedgehog tenrec)	ASM31398v2	No	2.95	2.61	43.01	7,490	277,896	54.42	20.43
Elephantulus edwardii	Cape elephant shrew	EleEdw1.0	No	3.84	3.12	40.30	8,768	288,162	15.01	24.22
Chrysochloris asiatica	Cape golden mole	ChrAsi1.0	No	4.21	3.36	40.01	20,500	391,344	13.47	19.63

Table S3 | BUSCO evaluation of afrotherian genome assemblies

For consistency, all genome assemblies were examined using the same version and library of BUSCO (5.0.0_cv1 with the 9,226-gene mammalia_odb10 data set). For additional assembly information, please see Table S2. Assemblies with the suffix _HiC were generated by DNA Zoo, the remainder are assemblies available via NCBI.

Afrotheria family	Latin name	Common name	Assembly	Complete BUSCOs (C)	Complete and single-copy BUSCOs (S)	Complete and duplicated BUSCOs (D)	Fragmented BUSCOs (F)	Missing BUSCOs (M)
Dugongidae	*Dugong dugon*	Dugong	Ddugon_BGI	8,712 (94.4%)	8,645	67	156	358
Dugongidae	*Dugong dugon*	Dugong	Ddugon_HiC	8,637 (93.6%)	8,564	73	195	394
Dugongidae	*Dugong dugon*	Dugong	GCA_030035595.1	8,875 (96.2%)	8,790	85	72	279
Dugongidae	*Dugong dugon*	Dugong	GCA_905400935.1	7,996 (86.6%)	7,900	96	460	770
Dugongidae	*Dugong dugon*	Dugong	GCA_015147995.1	5,843 (63.3%)	5,780	63	1,221	2,162
Dugongidae	*Hydrodamalis gigas*	Steller's sea cow	H_Gigas_1.0	1,288 (14.0%)	1,269	19	1,221	6,717
Trichechidae	*Trichechus manatus*	West Indian manatee	TriManLat1.0_HiC	8,682 (94.1%)	8,607	75	174	370
Trichechidae	*Trichechus manatus*	West Indian manatee	ASM3001377v1	8,490 (92.1%)	8,410	80	285	451
Elephantidae	*Elephas maximus*	Asian elephant	Elephas_maximus_HiC	8,483 (91.9%)	8,407	76	297	446
Procaviidae	*Procavia capensis*	Rock hyrax	Pcap_2.0_HiC	8,632 (93.6%)	8,567	65	167	427
Orycteropodidae	*Orycteropus afer*	Aardvark	OryAfe1.0_HiC	8,654 (93.8%)	8,582	72	185	387
Tenrecidae	*Echinops telfairi*	Lesser hedgehog tenrec	ASM31398v2	8,308 (90.0%)	8,151	157	301	617
Macroscelididae	*Elephantulus edwardii*	Cape elephant shrew	EleEdw1.0	8,727 (94.6%)	8,035	692	139	360
Chrysochloridae	*Chrysochloris asiatica*	Cape golden mole	ChrAsi1.0	8,853 (95.9%)	8,591	262	132	241

Table S4 | BUSCO evaluation of afrotherian gene sets

For consistency, all genome assemblies were examined using the same version and library of BUSCO (5.0.0_cv1 with the 9,226-gene mammalia_odb10 data set). Ddugon_BGI denotes the assembly generated in our study. Assemblies with the suffix _HiC were generated by DNA Zoo, the remainder are assemblies available via NCBI. Assembly Dugong_dugon_HiC is denoted 'Ddugon_DNAzoo' elsewhere in this manuscript.

Afrotheria family	Latin name	Common name	Assembly	Complete BUSCOs (C)	Complete and single-copy BUSCOs (S)	Complete and duplicated BUSCOs (D)	Fragmented BUSCOs (F)	Missing BUSCOs (M)
Dugongidae	*Dugong dugon*	Dugong	Ddugon_BGI	8,458 (91.7%)	8,410	48	300	468
Dugongidae	*Dugong dugon*	Dugong	Ddugon_HiC	7,407 (80.3%)	7,367	40	766	1,053
Trichechidae	*Trichechus manatus*	West Indian manatee	TriManLat1.0_HiC	7,358 (79.8%)	7,316	42	784	1,084
Elephantidae	*Elephas maximus*	Asian elephant	Elephas_maximus_HiC	7,274 (78.9%)	7,274	35	857	1,095
Procaviidae	*Procavia capensis*	Rock hyrax	Pcap_2.0_HiC	6,562 (71.2%)	6,520	42	867	1,797
Orycteropodidae	*Orycteropus afer*	Aardvark	OryAfe1.0_HiC	6,892 (74.7%)	6,860	32	962	1,372
Tenrecidae	*Echinops telfairi*	Lesser hedgehog tenrec	ASM31398v2	8,608 (93.3%)	5,901	2,707	276	342
Macroscelididae	*Elephantulus edwardii*	Cape elephant shrew	EleEdw1.0	8,904 (96.5%)	8,904	1,283	106	216
Chrysochloridae	*Chrysochloris asiatica*	Cape golden mole	ChrAsi1.0	9,056 (98.2%)	7,750	1,306	88	82

Table S5 | STRING literature mining enrichment of genes with inactivating mutations in sirenians

Gene set interrogated using STRING v12.0, which includes 'Reference publications' (i.e., publications with PubMed abstracts up to August 2022 and the PMC Open Access Subset up to April 2022).

term ID	term description	observed gene count	background gene count	strength	false discovery rate	matching proteins
PMID:35758554	(2022) Cetacean epidermal specialization: A review.	5	44	2.2	0.00073	ALOX15,TCHHL1,KLK8,AWAT2,TCHH

Table S6 | List of genes under selective sweep between dugong populations north and south of the Whitsundays Islands.

Genes were identified using XP-EHH, XP-CLR, π, and F_{ST}). Gene models with an XP prefix refers to NCBI GenBank annotations; evm.model, initial dugong gene models; homol.model, gene models obtained by adding additional homolog protein data. SIFT denotes, Sorting Intolerant From Tolerant; PolyPhen-2, Polymorphism Phenotyping-2 (results only available for human orthologs); N/A, not applicable.

Chromosome	Start	End	Gene model	Description	AA residue change (dugong protein)	AA residue change (human protein)	Functional impact of SNP
18	24,549,953	24,551,831	XP_003418414	CLPX caseinolytic mitochondrial matrix peptidase chaperone subunit X	I197T	I201T	SIFT: Tolerated (0.33); PolyPhen-2: Benign (0.276)
18	25,202,082	25,539,778	XP_007456442	putative V-set and immunoglobulin domain-containing-like protein IGHV4OR15-8-like	R52S	No ortholog	SIFT: Tolerated (0.33); PolyPhen2: N/A (no human ortholog)
18	25,333,034	25,333,399	evm.model.chr19.127	K06856 immunoglobulin heavy chain			
18	25,583,962	25,585,326	homol.model_1203	NUP42; nucleoporin NUP42 isoform X2			
18	26,350,303	26,411,351	evm.model.chr19.133	K06856 immunoglobulin heavy chain			

SUPPLEMENTARY REFERENCES

1. Pardini, A.T. *et al.* Chromosome painting among Proboscidea, Hyracoidea and Sirenia: support for Paenungulata (Afrotheria, Mammalia) but not Tethytheria. *Proc Biol Sci* **274**, 1333-40 (2007).
2. Le Duc, D. *et al.* Genomic basis for skin phenotype and cold adaptation in the extinct Steller's sea cow. *Sci Adv* **8**, eabl6496 (2022).
3. Rhie, A. *et al.* Towards complete and error-free genome assemblies of all vertebrate species. *Nature* **592**, 737-746 (2021).
4. Baker, D.N. *et al.* A chromosome-level genome assembly for the dugong (Dugong dugon). *J Hered* (2024).
5. Foote, A.D. *et al.* Convergent evolution of the genomes of marine mammals. *Nat Genet* **47**, 272-5 (2015).
6. Sharko, F.S. *et al.* Steller's sea cow genome suggests this species began going extinct before the arrival of Paleolithic humans. *Nature communications* **12**, 1-8 (2021).
7. Springer, M.S. Afrotheria. *Curr Biol* **32**, R205-R210 (2022).
8. Meredith, R.W. *et al.* Impacts of the Cretaceous Terrestrial Revolution and KPg extinction on mammal diversification. *Science* **334**, 521-4 (2011).
9. Nishihara, H., Hasegawa, M. & Okada, N. Pegasoferae, an unexpected mammalian clade revealed by tracking ancient retroposon insertions. *Proc Natl Acad Sci U S A* **103**, 9929-34 (2006).
10. Fernando, P. *et al.* DNA analysis indicates that Asian elephants are native to Borneo and are therefore a high priority for conservation. *PLoS Biol* **1**, E6 (2003).
11. Schwartz, G.T., Rasmussen, D.T. & Smith, R.J. Body-size diversity and community structure of fossil hyracoids. *Journal of Mammalogy* **76**, 1088-1099 (1995).
12. van der Valk, T. *et al.* Evolutionary consequences of genomic deletions and insertions in the woolly mammoth genome. *iScience* **25**, 104826 (2022).
13. Lynch, V.J. *et al.* Elephantid Genomes Reveal the Molecular Bases of Woolly Mammoth Adaptations to the Arctic. *Cell Rep* **12**, 217-28 (2015).
14. Christmas, M.J. *et al.* Evolutionary constraint and innovation across hundreds of placental mammals. *Science* **380**, eabn3943 (2023).
15. Liu, G.M. *et al.* Improved mammalian family phylogeny using gap-rare multiple sequence alignment: A timetree of extant placentals and marsupials. *Zool Res* **44**, 1064-1079 (2023).
16. Gheerbrant, E. Paleocene emergence of elephant relatives and the rapid radiation of African ungulates. *Proc Natl Acad Sci U S A* **106**, 10717-21 (2009).
17. Mason, V.C. *et al.* Genomic analysis reveals hidden biodiversity within colugos, the sister group to primates. *Sci Adv* **2**, e1600633 (2016).
18. Springer, M.S., Molloy, E.K., Sloan, D.B., Simmons, M.P. & Gatesy, J. ILS-Aware Analysis of Low-Homoplasy Retroelement Insertions: Inference of Species Trees and Introgression Using Quartets. *J Hered* **111**, 147-168 (2020).
19. Feng, S. *et al.* Incomplete lineage sorting and phenotypic evolution in marsupials. *Cell* **185**, 1646-1660 e18 (2022).

20. Doronina, L., Reising, O., Clawson, H., Ray, D.A. & Schmitz, J. True Homoplasy of Retrotransposon Insertions in Primates. *Syst Biol* **68**, 482-493 (2019).
21. Schull, J.K., Turakhia, Y., Hemker, J.A., Dally, W.J. & Bejerano, G. Champagne: Automated Whole-Genome Phylogenomic Character Matrix Method Using Large Genomic Indels for Homoplasy-Free Inference. *Genome Biol Evol* **14**(2022).
22. Marsh, H., O'Shea, T.J. & Reynolds III, J.E. *Ecology and conservation of the Sirenia: dugongs and manatees*, (Cambridge University Press, 2012).
23. Gallivan, G.J., Kanwisher, J.W. & Best, R.C. Heart rates and gas exchange in the Amazonian manatee (Trichechus inunguis) in relation to diving. *J Comp Physiol B* **156**, 415-23 (1986).
24. Madjene, C., Boutigny, A., Bouton, M.C., Arocas, V. & Richard, B. Protease Nexin-1 in the Cardiovascular System: Wherefore Art Thou? *Front Cardiovasc Med* **8**, 652852 (2021).
25. Crump, S.M. & Abbott, G.W. Arrhythmogenic KCNE gene variants: current knowledge and future challenges. *Front Genet* **5**, 3 (2014).
26. Storlund, R.L., Rosen, D.A.S. & Trites, A.W. Electrocardiographic Scaling Reveals Differences in Electrocardiogram Interval Durations Between Marine and Terrestrial Mammals. *Front Physiol* **12**, 690029 (2021).
27. Siegal-Willott, J. *et al.* Electrocardiography in two subspecies of manatee (Trichechus manatus latirostris and T. m. manatus). *J Zoo Wildl Med* **37**, 447-53 (2006).
28. Waycott, M., Biffin, E. & Les, D.H. Systematics and evolution of Australian seagrasses in a global context. in *Seagrasses of Australia: Structure, Ecology and Conservation* 129-154 (Springer, 2018).
29. Best, R.C. Foods and feeding habits of wild and captive Sirenia. *Mammal Review* **11**, 3-29 (1981).
30. Lanyon, J.M. & Sanson, G.D. Mechanical disruption of seagrass in the digestive tract of the dugong. *Journal of Zoology* **270**, 277-289 (2006).
31. Lanyon, J.M. & Sanson, G.D. Degenerate dentition of the dugong (Dugong dugon), or why a grazer does not need teeth: morphology, occlusion and wear of mouthparts. *Journal of Zoology* **268**, 133-152 (2006).
32. Lanyon, J. & Marsh, H. Digesta passage times in the dugong. *Australian Journal of Zoology* **43**, 119-127 (1995).
33. Eigeland, K.A. *et al.* Bacterial community structure in the hindgut of wild and captive dugongs (Dugong dugon). *Aquatic Mammals* **38**(2012).
34. Sharma, V. & Hiller, M. Losses of human disease-associated genes in placental mammals. *NAR Genom Bioinform* **2**, lqz012 (2020).
35. Emerling, C.A., Delsuc, F. & Nachman, M.W. Chitinase genes (CHIAs) provide genomic footprints of a post-Cretaceous dietary radiation in placental mammals. *Sci Adv* **4**, eaar6478 (2018).
36. Potter, J.H.T. *et al.* Dietary Diversification and Specialization in Neotropical Bats Facilitated by Early Molecular Evolution. *Mol Biol Evol* **38**, 3864-3883 (2021).
37. Newman, J. *et al.* Low activities of digestive enzymes in the guts of herbivorous grouse (Aves: Tetraoninae). *Journal of Ornithology* **162**, 477-485 (2021).

38. Sabat, P., Lagos, J.A. & Bozinovic, F. Test of the adaptive modulation hypothesis in rodents: dietary flexibility and enzyme plasticity. *Comp Biochem Physiol A Mol Integr Physiol* **123**, 83-7 (1999).
39. Ortiz, R.M., Mackenzie, D.S. & Worthy, G.A. Thyroid hormone concentrations in captive and free-ranging West Indian manatees (Trichechus manatus). *Journal of Experimental Biology* **203**, 3631-3637 (2000).
40. Eckhart, L. & Zeeuwen, P. The skin barrier: Epidermis vs environment. *Exp Dermatol* **27**, 805-806 (2018).
41. Martins, A.F. *et al.* Locally-curved geometry generates bending cracks in the African elephant skin. *Nat Commun* **9**, 3865 (2018).
42. Horgan, P., Booth, D., Nichols, C. & Lanyon, J.M. Insulative capacity of the integument of the dugong (Dugong dugon): thermal conductivity, conductance and resistance measured by in vitro heat flux. *Marine biology* **161**, 1395-1407 (2014).
43. Chernova, O., Kiladze, A. & Shpak, O. Papillomatous Skin Netting of Cetaceans (Cetacea: Delphinapterus leucas, Balaena mysticetus, and Eschrichtius robustus) and Sirens (Sirenia: Trichechus manatus and Dugong dugon). in *Doklady Biological Sciences* Vol. 481 150-156 (Springer, 2018).
44. Ehrlich, F. *et al.* Differential Evolution of the Epidermal Keratin Cytoskeleton in Terrestrial and Aquatic Mammals. *Mol Biol Evol* **36**, 328-340 (2019).
45. Sun, X. *et al.* Comparative genomics analyses of alpha-keratins reveal insights into evolutionary adaptation of marine mammals. *Front Zool* **14**, 41 (2017).
46. Huelsmann, M. *et al.* Genes lost during the transition from land to water in cetaceans highlight genomic changes associated with aquatic adaptations. *Sci Adv* **5**, eaaw6671 (2019).
47. Hecker, N., Sharma, V. & Hiller, M. Transition to an Aquatic Habitat Permitted the Repeated Loss of the Pleiotropic KLK8 Gene in Mammals. *Genome Biol Evol* **9**, 3179-3188 (2017).
48. Uhlen, M. *et al.* Proteomics. Tissue-based map of the human proteome. *Science* **347**, 1260419 (2015).
49. Lopes-Marques, M. *et al.* Complete Inactivation of Sebum-Producing Genes Parallels the Loss of Sebaceous Glands in Cetacea. *Mol Biol Evol* **36**, 1270-1280 (2019).
50. Zhang, X. *et al.* Parallel Independent Losses of G-Type Lysozyme Genes in Hairless Aquatic Mammals. *Genome Biol Evol* **13**(2021).
51. Berta, A., Sumich, J.L. & Kovacs, K.M. Chapter 7 - Integumentary and Sensory Systems. in *Marine Mammals (Third Edition)* (eds. Berta, A., Sumich, J.L. & Kovacs, K.M.) 169-210 (Academic Press, San Diego, 2015).
52. Rodrigues, F.R., da Silva, V.M.F. & Marques Barcellos, J.F. The mammary glands of the Amazonian manatee, Trichechus inunguis (Mammalia: Sirenia): morphological characteristics and microscopic anatomy. *The anatomical record* **297**, 1532-1535 (2014).
53. Liu, J. *et al.* Differential MC5R loss in whales and manatees reveals convergent evolution to the marine environment. *Dev Genes Evol* **232**, 81-87 (2022).
54. Springer, M.S. & Gatesy, J. Evolution of the MC5R gene in placental mammals with evidence for its inactivation in multiple lineages that lack sebaceous glands. *Mol Phylogenet Evol* **120**, 364-374 (2018).

55. Springer, M.S. *et al.* Genomic and anatomical comparisons of skin support independent adaptation to life in water by cetaceans and hippos. *Curr Biol* **31**, 2124-2139 e3 (2021).
56. Zhou, M., Wu, T., Chen, Y., Xu, S. & Yang, G. Functional Attenuation of UCP1 as the Potential Mechanism for a Thickened Blubber Layer in Cetaceans. *Mol Biol Evol* **39**(2022).
57. Hashimoto, O. *et al.* Brown adipose tissue in cetacean blubber. *PLoS One* **10**, e0116734 (2015).
58. Ikeda, K. & Yamada, T. UCP1 Dependent and Independent Thermogenesis in Brown and Beige Adipocytes. *Front Endocrinol (Lausanne)* **11**, 498 (2020).
59. Iwen, K.A., Oelkrug, R. & Brabant, G. Effects of thyroid hormones on thermogenesis and energy partitioning. *J Mol Endocrinol* **60**, R157-R170 (2018).
60. Robeck, T.R. *et al.* Thyroid hormone concentrations associated with age, sex, reproductive status and apparent reproductive failure in the Amazon river dolphin (Inia geoffrensis). *Conserv Physiol* **7**, coz041 (2019).
61. Yuan, Y. *et al.* Comparative genomics provides insights into the aquatic adaptations of mammals. *Proc Natl Acad Sci U S A* **118**(2021).
62. Mendes, T., Silva, L., Almeida, D. & Antunes, A. Neofunctionalization of the UCP1 mediated the non-shivering thermogenesis in the evolution of small-sized placental mammals. *Genomics* **112**, 2489-2498 (2020).
63. Gaudry, M.J. *et al.* Inactivation of thermogenic UCP1 as a historical contingency in multiple placental mammal clades. *Sci Adv* **3**, e1602878 (2017).
64. McGaugh, S. & Schwartz, T.S. Here and there, but not everywhere: repeated loss of uncoupling protein 1 in amniotes. *Biol Lett* **13**(2017).
65. Favilla, A.B. & Costa, D.P. Thermoregulatory strategies of diving air-breathing marine vertebrates: a review. *Frontiers in Ecology and Evolution* **8**, 555509 (2020).
66. Gaudry, M.J., Fyda, T.J. & Jastroch, M. Evolution of pinniped UCP1 is not linked to aquatic life but to neonatal thermogenesis and body size. *Proc Natl Acad Sci U S A* **119**(2022).
67. Yuan, Y. *et al.* Reply to Gaudry et al.: Cross-validation is necessary for the identification of pseudogenes. *Proc Natl Acad Sci U S A* **119**(2022).
68. Gaudry, M.J. *et al.* Terrestrial Birth and Body Size Tune UCP1 Functionality in Seals. *Mol Biol Evol* **41**(2024).
69. Albalat, R. & Canestro, C. Evolution by gene loss. *Nat Rev Genet* **17**, 379-91 (2016).
70. Weijs, L., Leusch, F. & Covaci, A. Concentrations of legacy persistent organic pollutants and naturally produced MeO-PBDEs in dugongs (Dugong dugon) from Moreton Bay, Australia. *Chemosphere* **229**, 500-508 (2019).
71. Meyer, W.K. *et al.* Ancient convergent losses of Paraoxonase 1 yield potential risks for modern marine mammals. *Science* **361**, 591-594 (2018).
72. Graham, A.M. *et al.* Reduction of Paraoxonase expression followed by inactivation across independent semiaquatic mammals suggests stepwise path to pseudogenization. *Mol Biol Evol* **40**(2023).

73. Paterson, R.S., Rybczynski, N., Kohno, N. & Maddin, H.C. A total evidence phylogenetic analysis of pinniped phylogeny and the possibility of parallel evolution within a monophyletic framework. *Frontiers in Ecology and Evolution* **7**, 457 (2020).
74. Zhao, B., Bie, J., Wang, J., Marqueen, S.A. & Ghosh, S. Identification of a novel intracellular cholesteryl ester hydrolase (carboxylesterase 3) in human macrophages: compensatory increase in its expression after carboxylesterase 1 silencing. *Am J Physiol Cell Physiol* **303**, C427-35 (2012).
75. Sanghani, S.P. *et al.* Hydrolysis of irinotecan and its oxidative metabolites, 7-ethyl-10-[4-N-(5-aminopentanoic acid)-1-piperidino] carbonyloxycamptothecin and 7-ethyl-10-[4-(1-piperidino)-1-amino]-carbonyloxycamptothecin, by human carboxylesterases CES1A1, CES2, and a newly expressed carboxylesterase isoenzyme, CES3. *Drug Metab Dispos* **32**, 505-11 (2004).
76. Lian, J., Nelson, R. & Lehner, R. Carboxylesterases in lipid metabolism: from mouse to human. *Protein Cell* **9**, 178-195 (2018).
