# Supplementary material for: Sirenian genomes illuminate the evolution of fully aquatic species within the mammalian superorder afrotheria
Source: Nat Commun. 2024 Jul 2;15:5568. doi: 10.1038/s41467-024-49769-x (PMC11219930; doi:10.1038/s41467-024-49769-x)
Supplement: Supplementary file 3 — Description of Additional Supplementary Files [file 41467_2024_49769_MOESM3_ESM.pdf]

Description of Additional Supplementary Files

File Name: Supplementary Data 1

Description: Positively selected genes (PSG) were identified using PAML with branch-site models. *P*-value denotes a **one-sided** χ^2-test statistic and adjusted P-value its multiple testing correction using the Benjamini-Hochberg method. A *P*-value or adjusted *P*-value of 0 indicates a number below 2.22×10^{-16}.

File Name: Supplementary Data 2

Description: KOBAS functional term enrichment of positively selected genes at the ancestral branch of Sirenia. *P*-value denotes a hypergeometric test (i.e., one-sided Fisher's exact test); Corrected *P*-value, multiple testing correction using the Benjamini-Hochberg method; the prefix 'hsa', KEGG (Kyoto Encyclopedia of Genes and Genomes) Pathways; the prefix 'GO', gene ontology terms.

File Name: Supplementary Data 3

Description: Rapidly evolving genes (REGs) were identified using PAML with branch-site models. *P*-value denotes a **one-sided** χ^2-test statistic and adjusted *P*-value its multiple testing correction using the Benjamini-Hochberg method. A *P*-value or adjusted *P*-value of 0 indicates a number below 2.22×10^{-16}.

File Name: Supplementary Data 4

Description: KOBAS functional term enrichment of rapidly evolving genes at the ancestral branch of Sirenia. *P*-value denotes a hypergeometric test (i.e., one-sided Fisher's exact test); Corrected *P*-value, multiple testing correction using the Benjamini-Hochberg method; the prefix 'hsa', KEGG (Kyoto Encyclopedia of Genes and Genomes) Pathways; the prefix 'GO', gene ontology terms.

File Name: Supplementary Data 5

Description: Sirenian-specific amino acid substitutions in thyroid hormone pathway and circadian clock genes. Potential functional effects of substitutions were predicted using PolyPhen-2 and SIFT. SIFT denotes, Sorting Intolerant From Tolerant; PolyPhen-2, Polymorphism Phenotyping-2 (results only available for human orthologs).

File Name: Supplementary Data 6

Description: Gene families expanded and contracted in sirenian genomes. Cdid, *Choloepus didactylus*; Oafe, *Orycteropus afer*; Eedw, *Elephantulus edwardii*; Pcap, *Procavia capensis*; Casi, *Chrysochloris asiatica*; Emax, *Elephas maximus*; Etel, *Echinops telfairi*; Ddug, *Dugong dugon*; Tman, *Trichechus manatus*. Nodes correspond to the NEXUS tree

((((Emax<0>,Pcap<2>)<1>,(Tman<4>,Ddug<6>)<5>)<3>,((Eedw<8>,(Etel<10>,Casi<12>)<11>)<9>,Oafe<14>)<13>)<7>,Cdid<16>)<15>.

File Name: Supplementary Data 7

Description: KOBAS functional term enrichment of expanded or contracted sirenian gene families.

P-value denotes a hypergeometric test (i.e., one-sided Fisher's exact test); Corrected *P*-value, multiple testing correction using the Benjamini-Hochberg method; the prefix 'hsa', KEGG (Kyoto Encyclopedia of Genes and Genomes) Pathways; the prefix 'GO', gene ontology terms.

File Name: Supplementary Data 8

Description: Genes with inactivating mutations in sirenians. Please note that stop codon of KCNK18 residue 462 in West Indian manatee and Steller's sea cow, but not dugong, presumably arose independently because the site is preceded by internal deletions in Dugongidae (Steller's sea cow and dugong) but not in the West Indian manatee.

File Name: Supplementary Data 9

Description: Statistics of dugong whole-genome resequencing samples. QLD denotes Queensland, Australia; NSW, New South Wales, Australia; WA, Western Australia, Australia. Samples SRR17870680, ERR5621402, and DRR251525 were obtained from NCBI SRA. The Rx ratio denotes the ratio of sequence reads that align to the X chromosome to reads that align to autosomal chromosomes and allows for sex determination of a sample (ca. 1.0 for females and 0.5 for males). Rx.CI denotes the Rx ratio 95% Confidence Interval upper and lower bound. Clean reads refer to the number of reads processed (removal of adaptors sequences and low-quality base calls) using SOAPnuke and Flexbar; mapping rate, percentage of reads that mapped to the dugong genome. Sequencing depth (average coverage across the genome) was calculated using mosdepth after downstream BAM file filtering to remove PCR duplicates and reads with a quality score below 30 (Q30). For comparative analyses, all samples were to downsampled to ~10× coverage.

File Name: Supplementary Data 10

Description: Patterson's *D* (ABBA-BABA) and *f4*-ratio statistics for all possible dugong population trios. Statistics were calculated using Dsuite. The West Indian manatee was specified as the outgroup population. The *D* statistic is calculated as the difference in the number of ABBA and BABA sites relative to the total number of sites examined. Dsuite uses jackknifing of the null hypothesis that no introgression has occurred (*D* statistic = 0) to calculate a *P*-value for each possible trio of populations. Dsuite also calculates the admixture fraction, or *f4*-ratio, which represents the covariance of allele frequency differences between P1 and P2 and between P3 and P4. If no introgression has occurred since P1 and P2 split from P3 and P4, then *f4* = 0. If the *f4* statistic is positive, this suggests a discordant tree topology indicative of introgression.

File Name: Supplementary Data 11

Description: Runs of homozygosity (ROHs) statistics of dugong whole-genome resequencing samples. QLD denotes Queensland, Australia; NSW, New South Wales, Australia; WA, Western Australia, Australia. Samples SRR17870680 (Exmouth Gulf, WA), ERR5621402 (Coogee Beach, NSW), and DRR251525 (Okinawa, Japan) were obtained from NCBI SRA.
