# Supplementary material for: Sirenian genomes illuminate the evolution of fully aquatic species within the mammalian superorder afrotheria
Source: Nat Commun. 2024 Jul 2;15:5568. doi: 10.1038/s41467-024-49769-x (PMC11219930; doi:10.1038/s41467-024-49769-x)
Supplement: Supplementary file 15 — Reporting Summary [file 41467_2024_49769_MOESM15_ESM.pdf]

nature portfolio

Corresponding author(s): Guangyi Fan
Songhai Li
Inge Seim

Last updated by author(s): 2024/05/26

Reporting Summary

Nature Portfolio wishes to improve the reproducibility of the work that we publish. This form provides structure for consistency and transparency in reporting. For further information on Nature Portfolio policies, see our Editorial Policies and the Editorial Policy Checklist.

Statistics

For all statistical analyses, confirm that the following items are present in the figure legend, table legend, main text, or Methods section.

n/a	Confirmed	
☐	☒	The exact sample size (*n*) for each experimental group/condition, given as a discrete number and unit of measurement
☐	☒	A statement on whether measurements were taken from distinct samples or whether the same sample was measured repeatedly
☐	☒	The statistical test(s) used AND whether they are one- or two-sided<br>*Only common tests should be described solely by name; describe more complex techniques in the Methods section.*
☒	☐	A description of all covariates tested
☐	☒	A description of any assumptions or corrections, such as tests of normality and adjustment for multiple comparisons
☐	☒	A full description of the statistical parameters including central tendency (e.g. means) or other basic estimates (e.g. regression coefficient) AND variation (e.g. standard deviation) or associated estimates of uncertainty (e.g. confidence intervals)
☐	☒	For null hypothesis testing, the test statistic (e.g. *F*, *t*, *r*) with confidence intervals, effect sizes, degrees of freedom and *P* value noted<br>*Give P values as exact values whenever suitable.*
☒	☐	For Bayesian analysis, information on the choice of priors and Markov chain Monte Carlo settings
☒	☐	For hierarchical and complex designs, identification of the appropriate level for tests and full reporting of outcomes
☒	☐	Estimates of effect sizes (e.g. Cohen's *d*, Pearson's *r*), indicating how they were calculated

Our web collection on statistics for biologists contains articles on many of the points above.

Software and code

Policy information about availability of computer code

Data collection

[Genome assembly]
stLFRdenovo pipeline (https://github.com/BGI-biotools/stLFRdenovo);GapCloser v1.12;Redundans v0.14a; HiC-Pro v3.2.0_devel;Juicebox v1.11.08

[Genome annotation]
BLAST;RepeatMasker v4.0.5 with Repbase database v16.02;RepeatModeler v1.1.0.4;LTRharvest v1.5.8;Tandem Repeat Finder v4.07;BLAT v0.36;GeneWise v2.4.1;AUGUSTUS v3.2.3;GENSCAN v1.0;GlimmerHMM v3.0.1;Flexbar v3.4.0;SortMeRNA v2.1b 123 with SILVA ribosomal database v119;HISAT2 v2.1.0;SAMtools v1.9;TransDecoder v5.5.0;EvidenceModeler v1.1.1;BLAST;BUSCO v5.4.3 with mammalia_odb10 gene set

Data analysis

[Phylogeny and divergence time estimation]
OrthoFinder v2.5.4;PRANK v70427;Gblocks v0.91b;LASTZ v1.04.22;MULTIZ v11.2;phyloFit v1.4;MARS (Multiple circular sequence Alignment using Refined Sequences);RaxML v8.2.9;BUSCO v5.4.3 with mammalia_odb10 gene set;ASTRAL-III v5.6.2;PAML v4.8;CAFE v4.2

[Sirenian gene selection]
PAML v4.8;KOBAS v3.0;STRING v12.0

[Gene loss in the sirenian lineage]
BLAT v36;genBlastA v1.0.1;GeneWise v2.4.1;BLAST

[Sirenian lineage-specific amino acid changes]

FasParser v2;BLAST;SIFT v6.2.1;PolyPhen-2

[Whole-genome resequencing]
SOAPnuke v2.1.5;SAMtools v1.9;BLAST;bowtie2 v2.3.4.3;SAMtools v1.7; R script for inference of biological sex using the Rx approach (PMID IDs 27706187 and 32107273);mosdepth v0.3.3

[Identification and characterization of SNPs]
bowtie2 v2-2.2.5;BWA v0.7.12-r1039;SAMtools v1.2;Picard v1.54;GATK v4.1.2.0

[Analysis of population structure]
vcftools v0.1.16;Plink v1.90b6.6;ADMIXTURE v1.3.0;MEGA7;iTOL v6 (https://itol.embl.de);ggplot2 R package;dadi v2.1

[Estimation of genome heterozygosity and runs of homozygosity]
detectRUNS R package;FASTEPRR v2

[Identification of selective sweep regions]
vcftools v0.1.16;xpclr v1.1.2;PPP v0.1.12;Hapbin v1.3.0

[Demographic history inference]
PSMC v0.6.5-r67;SAMtools v1.9;BCFtools v1.4;SMC++ v1.15.5

For manuscripts utilizing custom algorithms or software that are central to the research but not yet described in published literature, software must be made available to editors and reviewers. We strongly encourage code deposition in a community repository (e.g. GitHub). See the Nature Portfolio guidelines for submitting code & software for further information.

Data

Policy information about availability of data

All manuscripts must include a data availability statement. This statement should provide the following information, where applicable:

- Accession codes, unique identifiers, or web links for publicly available datasets
- A description of any restrictions on data availability
- For clinical datasets or third party data, please ensure that the statement adheres to our policy

Dugong sequencing reads (including stLFR, Hi-C, RNA-seq, resequencing) and the Ddugon_BGI genome assembly are available at NCBI BioProject PRJNA1114306 [https://www.ncbi.nlm.nih.gov/bioproject/?term=PRJNA1114306]. Dugong SNP data in VCF format are available at the European Nucleotide Archive (ENA) and linked to the NCBI BioProject. Multiple sequence alignments (MSAs) of thyroid hormone pathway and circadian clock genes with sirenian-specific amino acid substitutions are available on FigShare [https://doi.org/10.6084/m9.figshare.23975559].

Research involving human participants, their data, or biological material

Policy information about studies with human participants or human data. See also policy information about sex, gender (identity/presentation), and sexual orientation and race, ethnicity and racism.

Reporting on sex and gender	N/A
Reporting on race, ethnicity, or other socially relevant groupings	N/A
Population characteristics	N/A
Recruitment	N/A
Ethics oversight	N/A

Note that full information on the approval of the study protocol must also be provided in the manuscript.

Field-specific reporting

Please select the one below that is the best fit for your research. If you are not sure, read the appropriate sections before making your selection.

☐ Life sciences ☐ Behavioural & social sciences ☒ Ecological, evolutionary & environmental sciences

For a reference copy of the document with all sections, see nature.com/documents/nr-reporting-summary-flat.pdf

Ecological, evolutionary & environmental sciences study design

All studies must disclose on these points even when the disclosure is negative.

Study description	This study includes the generation of a dugong reference genome from a fetal liver tissue sample, RNA-sequencing of the liver sample and a skin sample, and whole-genome resequencing of skin samples from 99 individuals.
Research sample	Fetal liver tissue (sample D201106) for reference genome sequencing was obtained from a recently deceased near-term female dugong fetus recovered from a cow that was hunted illegally in the Burrum Heads region of Hervey Bay Queensland in November 2020.<br><br>We also sampled 100 dugongs from seven geographic locations on the east coast of Australia: Airlie Beach (AB, n=3), Bowling Green Bay (BG, n=3), Clairview (CV, n=9), Great Sandy Strait (GS, n=23), Hervey Bay (HB, n=24), Moreton Bay (MB, n=32), and Torres Strait (D, n=6; prefix 'TS' elsewhere in the manuscript). Of these, 99 were subject to genome-sequencing and one Torres Strait sample to transcriptome sequencing (RNA-seq).
Sampling strategy	Skin samples was collected from the dorsum of wild free-swimming dugongs (94 individuals, i.e., all populations except Torres Strait) using a handheld scraper device. In the Torres Strait, skin was excised from fresh dugong carcasses post-hunt by local Traditional Owners at Mabiuag Island.<br><br>No statistical methods were used to predetermine sample size. The sample size per population is based on available samples.
Data collection	High-molecular weight DNA was extracted from a fetal liver sample (D201106) using a MagAttract HMW DNA Kit (QIAGEN). DNA quantity, purity and integrity were assessed by Qubit fluorometry (Invitrogen), Nanodrop spectrophotometry (Thermo Fisher Scientific), and pulse-field gel electrophoresis. Single-tube long fragment read (stLFR) libraries 100 were sequenced on a MGISEQ-2000 sequencer. Hi-C libraries were prepared from the same fetal liver sample. Hi-C data (200 Gb 150 bp paired-end reads) were generated on the BGISEQ-500 platform.<br><br>99 skin samples were sequenced on a DNBSEQ-G400 RS instrument by BGI-Australia to generate 100-bp paired end reads. We also obtained public sequencing data from Okinawa (Japan; DRR251525; sampled 17 November 2019), Coogee Beach (New South Wales, Australia; ERR5621402; sampled 25 November 2009) 66, and Exmouth Gulf (Western Australia, Australia; SRR17870680; sampled 3 June 2018). We obtained 101-bp paired-end WGS reads generated on the Illumina HiSeq 2000 platform (NCBI SRA SRR331137, SRR331139, and SRR331142) from a female West Indian manatee (Lorelei, born in captivity in Florida, USA) 163. The manatee reads were employed as outgroups in population genomics analyses. Raw data were filtered using SOAPnuke v2.1.5 101 to remove adapters and low-quality reads. For comparative analyses, all samples were down-sampled to ~10× coverage using SAMtools v1.9 126.<br><br>RNA from fetal liver (sample D201106) and skin (sample D110419) extracted using an RNeasy Mini Kit (QIAGEN) was sequenced on the BGISEQ-500 platform.<br><br>DNA from 99 dugongs sampled on the Australian east coast (see 'Sample collection and research ethics') was extracted using a QIAamp DNA Mini Kit (QIAGEN) and sequenced on a DNBSEQ-G400 RS instrument by BGI-Australia to generate 100-bp paired end reads.
Timing and spatial scale	The samples utilized in this study were obtained at multiple field trips by co-author Janet M. Lanyon in the last decade. The frequency and periodicity of sampling is not applicable to our study since genomic resequencing data does not vary with fine scale collection time.
Data exclusions	Raw genome resequencing data were filtered using SOAPnuke v2.1.5 101 to remove adapters and low-quality reads. For comparative analyses, all samples were down-sampled to ~10× coverage using SAMtools v1.9. GATK v4.1.2.0 was utilized to realign reads around InDels and detect SNPs. Briefly, we obtained the genomic variant call format (GVCF) in ERC mode based on read mapping with the parameters '-T HaplotypeCaller, -stand-call-conf 30.0 -ERC GVCF'. Joint variant calling was then conducted with the GATK CombineGVCFs module. Lastly, the GATK's VariantFiltration module was used for hard filtering with the parameters '--filter-name LowQualFilter --filterExpression QD < 2.0 || MQ < 40.0 || FS > 60.0 || ReadPosRankSum < -8.0 || MQRankSum < -12.5 || SOR > 3.0', as recommended by GATK. This process generated 61,741,769 SNPs.
Reproducibility	No attempts was performed to repeat the above experiments.
Randomization	Resequencing samples were not grouped prior to the PCA, admixture, and neighbor-joining tree analyses that assigned samples into population groups.
Blinding	With regards to dugong sampling, there is blinding because it is unknown if any samples will be collected any given day and if samples are collected, the sampled individuals are unknown. All sequencing was performed at the same facility BGI (Brisbane), with BGI technicians blind to the sample origin.

Did the study involve field work? ☒ Yes ☐ No

Field work, collection and transport

Field conditions	The liver sample for reference genome sequencing was transported frozen to The University of Queensland and dissected by J.M.L.<br><br>Skin samples for whole-genome resequencing (99) and RNA-sequencing (1) were stored in salt-saturated 20% dimethyl sulfoxide (DSMO) and frozen at -20 °C until further processing for resequencing.
Location	All samples were collected on the Australian east coast (Queensland). Airlie Beach (AB, n=3), Bowling Green Bay (BG, n=3), Clairview (CV, n=9), Great Sandy Strait (GS, n=23), Hervey Bay (HB, n=24), Moreton Bay (MB, n=32), and Torres Strait (D, n=5; prefix 'TS' used in the manuscript).
Access & import/export	The dugong skin samples were collected under the following permits issued to J.M.L.: The University of Queensland Animal Ethics Permits SBS/360/14, SBS/181/18, Scientific Purposes Permits WISP07255110 and WISP14654414, Moreton Bay Marine Parks Permit #QS2000 to #QS2010CV L228, Great Sandy Marine Parks Permit QS2010-GS043, and Great Barrier Reef Marine Park Permits #G07=23274:1 and G14/36987.1.
Disturbance	The skin sampling protocol we followed is minimally invasive, it only takes a small skin sample and the animal is not harassed for long periods of time. Therefore, the sampling activities that we performed are very unlikely to have caused any kind of disturbance of this natural system.

Reporting for specific materials, systems and methods

We require information from authors about some types of materials, experimental systems and methods used in many studies. Here, indicate whether each material, system or method listed is relevant to your study. If you are not sure if a list item applies to your research, read the appropriate section before selecting a response.

Materials & experimental systems

n/a	Involved in the study
☐	☒ Antibodies
☐	☒ Eukaryotic cell lines
☒	☐ Palaeontology and archaeology
☐	☒ Animals and other organisms
☒	☐ Clinical data
☒	☐ Dual use research of concern
☒	☐ Plants

Methods

n/a	Involved in the study
☒	☐ ChIP-seq
☒	☐ Flow cytometry
☒	☐ MRI-based neuroimaging

Antibodies

Antibodies used	Mouse monoclonal anti-HA (Proteintech cat. no 66006-2-Ig at 1:50000 dilution) or anti-FLAG (Proteintech cat. no 66008-4-Ig at 1:25000 dilution) antibodies, and an anti-mouse secondary antibody (Proteintech cat. no SA00001-1 at 1:6000 dilution).
Validation	• The mouse anti-FLAG antibody targets DYKDDDDK residues in WB, RIP, IP, IHC, IF, CoIP, ChIP, and ELISA applications (positive IP detected in transfected HEK-293 cells; 147 Publications). See https://www.ptgcn.com/products/Flag-tag-Antibody-66008-4-Ig.htm#tested-applications.<br>• The mouse anti-HA antibody targets HA YPYDVPDYA residues in WB, PLA, IP, IF, FC, CoIP, ChIP, and ELISA applications (positive IP detected in transfected HEK-293 cells, 169 Publications). See https://www.ptgcn.com/products/HA-Tag-Antibody-66006-2-Ig.htm.

Eukaryotic cell lines

Policy information about cell lines and Sex and Gender in Research

Cell line source(s)	HEK293T (human embryonic kidney, transformation and stable clone generation 293-derivative)
Authentication	None of the cell lines used were authenticated
Mycoplasma contamination	Cell lines were not tested for mycoplasma infection
Commonly misidentified lines (See ICLAC register)	None

Animals and other research organisms

Policy information about studies involving animals; ARRIVE guidelines recommended for reporting animal research, and Sex and Gender in Research

Laboratory animals	The study did not include laboratory animals
Wild animals	Dugongs were not captured in our study
Reporting on sex	The determination of age and sex were not relevant for our study. However, we have obtained sex information from our genome analysis, revealing that of the 99 samples employed in genome re-sequencing analysis 36.4% were males and 64.6% females
Field-collected samples	See 'Field collection' section above
Ethics oversight	Fieldwork was conducted under Australian research permits (see 'Access & import/export' above)

Note that full information on the approval of the study protocol must also be provided in the manuscript.

Plants

Seed stocks	N/A
Novel plant genotypes	N/A
Authentication	N/A
